# Wnt/beta-catenin signaling confers ferroptosis resistance by targeting GPX4 in gastric cancer

**DOI:** 10.1038/s41418-022-01008-w

**Published:** 2022-05-09

**Authors:** Yue Wang, Lixin Zheng, Wenjing Shang, Zongcheng Yang, Tongyu Li, Fen Liu, Wei Shao, Lin Lv, Li Chai, Lingxin Qu, Qing Xu, Jie Du, Xiuming Liang, Jiping Zeng, Jihui Jia

**Affiliations:** 1 Department of Microbiology/Key Laboratory for Experimental Teratology of the Chinese Ministry of Education, School of Basic Medical Science, Cheeloo College of Medicine, Shandong University, Jinan, Shandong PR China; 2 Key Laboratory of Infection and Immunity of Shandong Province, School of Basic Medical Science, Cheeloo College of Medicine, Shandong University, Jinan, Shandong PR China; 3 Shandong University-Karolinska Institute Collaborative Laboratory for Cancer Research, School of Basic Medical Science, Cheeloo College of Medicine, Shandong University, Jinan, Shandong PR China; 4 Biomolecular Medicine, Clinical Research Center, Department of Laboratory Medicine, Karolinska Institute, Stockholm, Sweden; 5 Department of Biochemistry and Molecular Biology, School of Basic Medical Sciences, Cheeloo College of Medicine, Shandong University, Jinan, Shandong PR China

**Keywords:** Oncogenes, Microbiology, Tumour biomarkers

## Abstract

The development of chemotherapy resistance is the most vital obstacle to clinical efficacy in gastric cancer (GC). The dysregulation of the Wnt/beta-catenin signaling pathway is critically associated with GC development and chemotherapy resistance. Ferroptosis is a form of regulated cell death, induced by an iron-dependent accumulation of lipid peroxides during chemotherapy. However, whether the Wnt/beta-catenin signaling directly controls resistance to cell death, remains unclear. Here, we show that the activation of the Wnt/beta-catenin signaling attenuates cellular lipid ROS production and subsequently inhibits ferroptosis in GC cells. The beta-catenin/TCF4 transcription complex directly binds to the promoter region of GPX4 and induces its expression, resulting in the suppression of ferroptotic cell death. Concordantly, TCF4 deficiency promotes cisplatin-induced ferroptosis in vitro and in vivo. Thus, we demonstrate that the aberrant activation of the Wnt/beta-catenin signaling confers ferroptosis resistance and suggests a potential therapeutic strategy to enhance chemo-sensitivity for advanced GC patients.

## Introduction

Gastric cancer (GC) is the fourth leading cause of cancer-related deaths worldwide [1]. As a class I carcinogen, helicobacter pylori is the greatest risk factor for gastric cancer [2–4]. Most GC patients are usually poorly prognosed, due to late diagnosis and poor response to available therapies [5]. Cisplatin-based chemotherapies remain the primary treatments for advanced GC [6, 7], but the development of chemotherapy resistance is the most vital challenge for clinical efficacy [8]. Therefore, further investigation of the mechanism of cisplatin resistance to enhance its tumor sensitivity is essential for improving the prognosis and survival of patients with advanced GC.

More than half of GC patients have a dysregulation of the Wnt/beta-catenin signaling, which is a major cause of gastric cancer development [9–11]. During the activation of Wnt/beta-catenin signaling, beta-catenin translocates from the cytoplasm to the nucleus, where it binds to the TCF/LEF family to regulate the transcription of Wnt-target genes [12, 13]. In the TCF/LEF family, TCF4 (also known as TCF7L2) is the most important transcription factor that acts as a switch of the Wnt/beta-catenin pathway [14–16]. Although a sustained activation of the Wnt/beta-catenin signaling is associated with chemotherapy resistance [17–19], the mechanism remains largely unexplored.

Resistance to cell death is a vital distinctive hallmark of cancer, by which GC cells resist therapy [20]. Ferroptosis is a novel form of regulated cell death, characterized by the accumulation of iron-dependent lipid peroxides [21–23]. Glutathione peroxidase 4 (GPX4) is a key regulator of ferroptosis where it utilizes reduced glutathione to convert lipid hydroperoxides to lipid alcohols, which mitigate lipid peroxidation and inhibit ferroptosis [24–26]. GPX4 increased expression in tumors significantly correlates with tumorigenesis and metastasis. GPX inhibition or ablation enhances lipid peroxidation chain reaction that induces ferroptosis in different cancer cell types [24]. Since ferroptosis can be induced by chemotherapy, its control is associated with tumor development and chemotherapy resistance [27–29]. However, whether Wnt/beta-catenin signaling regulates ferroptosis, remains elusive.

In the present study, we reveal that the activation of the Wnt/beta-catenin signaling attenuates cellular lipid ROS production, and subsequently, inhibits ferroptosis in GC cells. The beta-catenin/TCF4 transcription complex directly binds to the promoter region of GPX4 and induces its expression, resulting in the suppression of ferroptotic cell death. Additionally, TCF4 inhibition or knockout significantly enhanced tumor sensitivity to cisplatin in vitro and in vivo by triggering ferroptosis. Importantly, we demonstrate the mechanism by which Wnt/beta-catenin signaling controls ferroptosis to promote tumor chemotherapy resistance. Our work suggests a potential therapeutic strategy to enhance chemo-sensitivity for advanced GC patients.

## Results

### The Inhibition of the Wnt/beta-catenin signaling selectively enhances GC cells sensitivity to ferroptosis

**Fig. 1:**
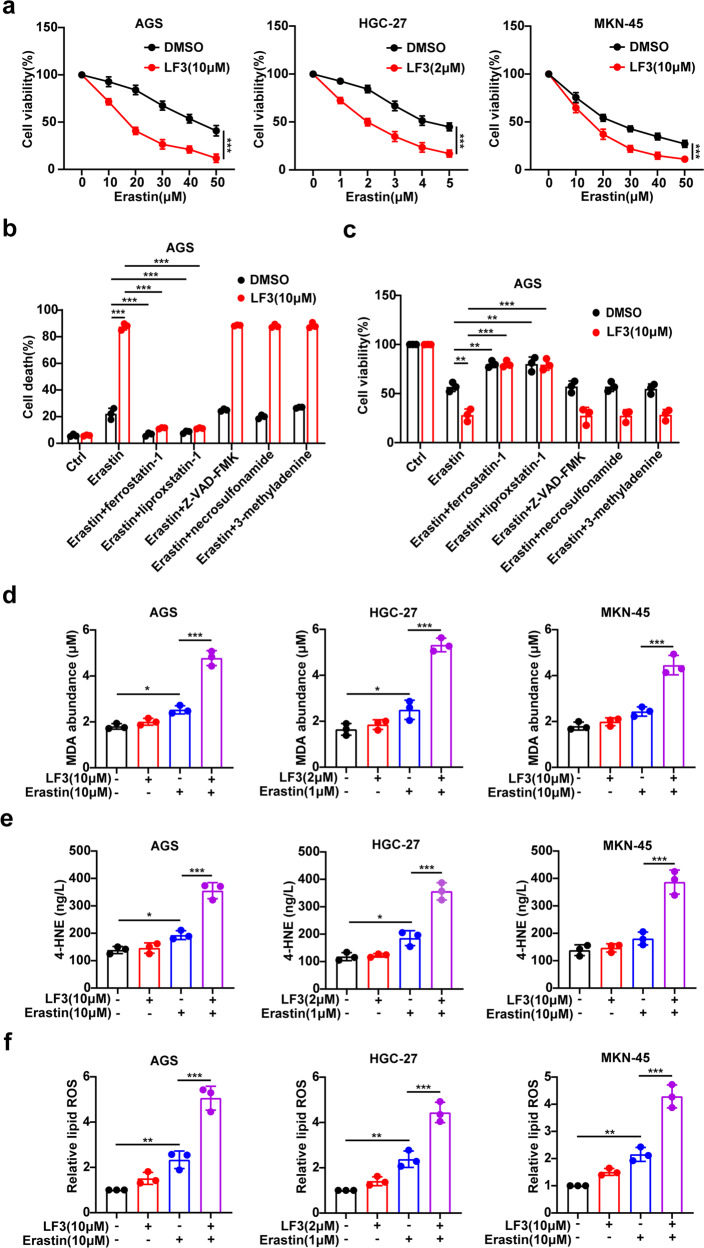
Inhibition of the Wnt/beta-catenin signaling enhances GC cells’ sensitivity to ferroptosis. **a** Cell viability of indicated GC cells following treatment with erastin in the absence or presence of LF3 (AGS and MKN-45 for 10 μΜ, HGC-27 for 2 μΜ) for 24 h. **b** Cell death measurement of AGS following treatment with erastin (20 μM) in the absence or presence of ferrostatin-1 (2 μM), liproxstatin-1 (1 μM), Z-VAD-FMK (10 μM), necrosulfonamide (0.5 μM), or 3-methyladenine (250 μM) for 24 h. **c** Cell viability of AGS treated with erastin (50 μM) in the absence or presence of ferrostatin-1 (2 μM), liproxstatin-1 (1 μM), Z-VAD-FMK (10 μM), necrosulfonamide (0.5 μM), or 3-methyladenine (250 μM) for 24 h. **d–f** MDA (**d**), 4-HNE (**e**), and lipid ROS (**f**) measurements in GC cells following treatment with erastin (AGS and MKN-45 for 10 μM and HGC-27 for 1 μΜ) and/or LF3 (AGS and MKN-45 for 10 μΜ, HGC-27 for 2 μΜ). Data are presented as the mean ± SD of three independent experiments. The *p*-values in panels **a**, **d**, **e**, **f** were calculated using two-way ANOVA. The *p*-values in panels **b**, **c** were calculated by one-way ANOVA. *P < 0.05, **P < 0.01, ***P < 0.001.

Cell death resistance is a distinctive hallmark of cancer, that confers GC cells resistance to therapy [20]. To determine whether the Wnt/beta-catenin signaling regulates resistance to cell death, we treated GC cells with LF3 and then stimulated them with inhibitors of different types of cell death. LF3 is a small molecule that inhibits Wnt/beta-catenin signals by disrupting the critical interaction between beta-catenin and the transcription factor TCF4 [30]. Interestingly, LF3 treatment selectively enhanced sensitivity to erastin-induced ferroptosis but did not affect actinomycin D-induced autophagy, TNFα-induced necroptosis, or H_2_O_2_-induced apoptosis (Fig. 1a and supplementary Fig. S1a-c). We further confirmed that erastin only induces ferroptosis and no other types of cell death in GC cells (Supplementary Fig. S1d). Moreover, erastin-induced cell death that is promoted by LF3, was significantly blocked by the ferroptosis inhibitor ferrostatin-1, and liproxstatin-1 but not by the apoptosis inhibitor Z-VAD-FMK, the necroptosis inhibitor necrosulfonamide, or autophagy inhibitor 3-methyladenine (Fig. 1b, c and Supplementary Fig. S1e). These results indicate that the inhibition of the Wnt/beta-catenin signaling suppresses GC cells resistance to cell death by selectively enhancing their sensitivity to ferroptosis.

Ferroptosis is a distinctive form of regulated cell death that is driven by a lethal accumulation of lipid peroxides in plasma membranes [22]. The BODIPY-C11 probe can specifically detect ferroptosis by determining the number of lipid peroxides in cellular membranes. Malondialdehyde (MDA) is the principal and most studied product of polyunsaturated fatty acid peroxidation, while 4-hydroxynonenal (4-HNE) is a key lipid peroxidation product that causes cellular dysfunction by forming adducts with proteins [31]. Therefore, we next used these compounds to verify if there was an elevation in the production of MDA, 4-HNE, and lipid peroxides in LF3 preincubated cells upon erastin treatment. The expressions of MDA, 4-HNE and lipid ROS were slightly increased in the LF3 or erastin treated group compared with the control group; however, they were markedly increased in LF3 preincubated cells upon erastin treatment (Fig. 1d–f). Taken together, these data indicate that the inhibition of the Wnt/beta-catenin signals, increases cellular lipid peroxidation, and promotes GC cells sensitivity to ferroptosis.

### TCF4 acts as a repressor of ferroptosis in GC cells

Following its activation, beta-catenin translocates into the nucleus and associates with the transcription factors TCF/LEF to promote the transcription of Wnt-target genes. As mentioned in the introduction, TCF4 is the main transcription effector in the Wnt/ beta-catenin signaling pathway [14, 32]. Further analyses in the TCGA and GEO database revealed that TCF4 expression was significantly upregulated in tumor tissues compared with that in normal tissue counterparts and both TCGA and histological subtypes (Supplementary Fig. S2a, b). Consistently, TCF4 expression was low in normal tissues and strikingly upregulated in GC tissues (Supplementary Fig. S2c-e). Kaplan–Meier analyses showed that a high-level expression of TCF4 significantly correlates with poor overall survival of GC patients (Supplementary Fig. S2f). These results strongly indicate that TCF4 has an oncogenic role in GC where it plays an important role in the Wnt/beta-catenin signaling.

**Fig. 2:**
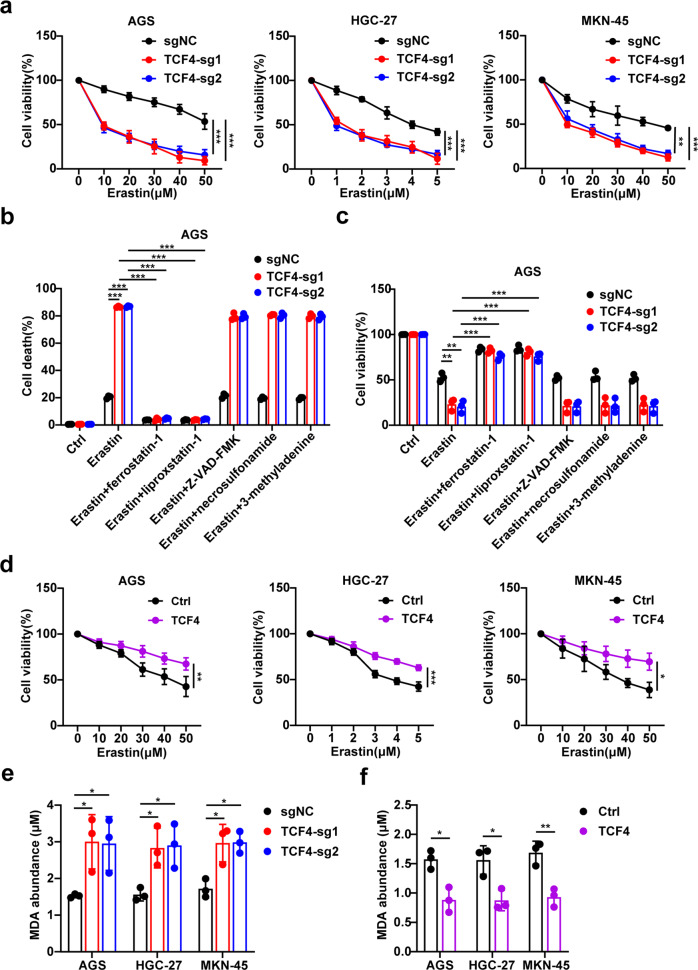
TCF4 acts as a repressor of ferroptosis in GC cells. **a** Cell viability of GC cells expressing sgNC or sg-TCF4 treated with different concentrations of erastin for 24 h. **b** Cell viability of GC cells transfected with control or TCF4-coding plasmid and treated with erastin for 24 h. **c** Cell death measurement of AGS expressing sgNC or sg-TCF4 treated with erastin (20 μM) in the absence or presence of ferrostatin-1 (2 μM), liproxstatin-1 (1 μM), Z-VAD-FMK (10 μM), necrosulfonamide (0.5 μM), or 3-methyladenine (250 μM) for 24 h. **d** Cell viability of AGS expressing sgNC or sg-TCF4 treated with erastin (50 μM) in the absence or presence of ferrostatin-1 (2 μM), liproxstatin-1 (1 μM), Z-VAD-FMK (10 μM), necrosulfonamide (0.5 μM), or 3-methyladenine (250 μM) for 24 h. **e** MDA production in GC cells expressing sgNC or sg-TCF4. **f** MDA production in GC cells transfected with control or TCF4-coding plasmid. Data are presented as the mean ± SD of three independent experiments. The *p*-values in panels **a**–**d** were calculated by two-way ANOVA. The *p*-values in panel **e** were calculated by one-way ANOVA. The *p*-values in panle **f** were calculated by Student’s *t*-test and one-way ANOVA. *P < 0.05, **P < 0.01, ***P < 0.001.

To further confirm the effect of TCF4 in ferroptosis, we generated TCF4-knockout GC cells using two independent sgRNAs (Supplementary Fig. S2g). The depletion of TCF4 increased erastin-induced ferroptosis (Fig. 2a), which was rescued by treatments of the cells with ferroptosis inhibitor ferrostatin-1 and liproxstatin-1 but not with the apoptosis inhibitor Z-VAD-FMK, the necroptosis inhibitor necrosulfonamide, nor the autophagy inhibitor 3-methyladenine (Fig. 2b, c and Supplementary Fig. S2h, i). Conversely, TCF4 overexpressing GC cells were robustly resistant to erastin-induced ferroptosis (Fig. 2d). Moreover, TCF4-knockout GC cells exhibited an increase in MDA and 4-HNE production, while TCF4 overexpression decreased MDA and 4-HNE production (Fig. 2e, f and Supplementary Fig. S2j, k). Together, these findings indicate that the Wnt/beta-catenin signaling promotes TCF4 activation to inhibit ferroptosis.

### TCF4 negatively modulates ferroptosis by targeting GPX4

**Fig. 3:**
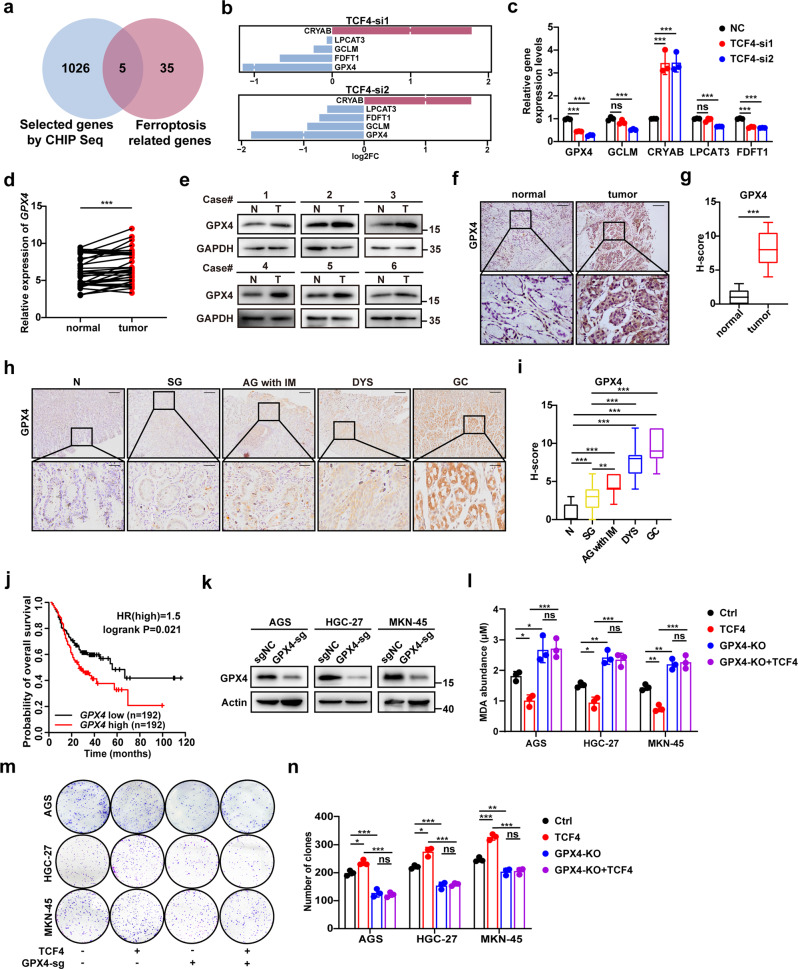
TCF4 negatively modulates ferroptosis through GPX4 targeting. **a** Venn diagram showing genes at the intersection of TCF4 bound promoters that were identified by ChIP-seq and previously reported ferroptosis-related genes. **b** Bar chart showing log2FC of the expression of five genes in TCF4 siRNA transfected HGC-27 cell. **c** Relative gene expression levels of GPX4, GCLM, CRYAB, LPCAT3, and FDFT1. **d** Q-PCR analysis of GPX4 mRNA expression in paired GC tissues and adjacent normal tissues (*n* = 34). **e** Protein expression of GPX4 in GC tissues (T) and adjacent normal tissues (N). **f**, **g** IHC staining (**f**) and H-score (**g**) for GPX4 in adjacent normal tissues (normal, *n* = 9) and GC tissues (tumor, *n* = 9). Scale bars: 200 μm (insets 50 μm). **h**, **i** IHC staining (**h**) and H-score (**i**) for GPX4 in N (normal tissues, *n* = 21), SG (superficial gastritis, *n* = 21), AG with IM (atrophic gastritis with intestinal metaplasia, *n* = 21), DYS (dysplasia, *n* = 25), and GC (*n* = 21) samples. Scale bars: 200 μm (insets 50 μm). **j** The overall survival for GC patients was analyzed using Kaplan–Meier curves (log-rank test; *n* = 384). **k** GPX4 protein expression in sgNC or sg-GPX4 expressing GC cells. **l** MDA production in GC cells expressing sgNC or sg-GPX4 and transfected with the TCF4-coding plasmid. **m**, **n** Colony formation of GC cells expressing sgNC or sg-GPX4 and transfected with the TCF4-coding plasmid. Data are presented as the mean ± SD of three independent experiments. The *p*-values in panels **c**, **i**, **l**, **n** were calculated by one-way ANOVA. The *p*-values in panels **d**, **g** were calculated by Student’s *t*-test. ns not significant, *P < 0.05, **P < 0.01, ***P < 0.001.

As a transcription factor, TCF4 promotes the transcription of its target genes by binding with their promoters; therefore, it was of interest to identify downstream transcriptional targets of TCF4. ChIP-seq assay showed that TCF4 bind to the promoters of 5 among 40 ferroptosis-related genes [33] in HGC-27 cells (Fig. 3a). GPX4 is a key regulator of ferroptosis, and thus, showed the most obvious decrease in TCF4-knockdown cells (Fig. 3b, c and Supplementary Fig. S3a–f). Further analyses of the TCGA and GEO database revealed that GPX4 expression was significantly upregulated in tumor tissues compared with their normal counterparts (Supplementary Fig. S3g–k). GPX4 protein and mRNA expression in human GC tissue samples were visibly increased compared with normal adjacent tissues (Fig. 3d, e). IHC staining showed an extremely low GPX4 expression in normal tissues and this expression was relatively low in superficial gastritis (SG), mildly upregulated in atrophic gastritis with intestinal metaplasia (AG with IM), moderately increased in dysplasia (DYS), and remarkably upregulated in GC tissues (Fig. 3f–i). Kaplan–Meier analyses showed that GPX4 high-level of expression significantly correlates with poor overall survival of GC patients (Fig. 3j).

Next, we investigated the biological role of GPX4 in tumorigenesis and metastasis. We found that GPX4 knockdown inhibited the DNA-replicating capacity of GC cells and cell viability (Supplementary Fig. S3l and Supplementary Fig. S4a, b). Cell migration and wound healing assays showed that GPX4 suppression markedly reduced invasion and migration in GC cells (Supplementary Fig. S4c, d).

To further explore the role of GPX4 in TCF4 negative regulation of ferroptosis, we generated GPX4-knockout GC cells (Fig. 3k). Our results showed that TCF4-mediated depletion of MDA and enhancement of tumorigenesis were relieved by GPX4 deficiency (Fig. 3l–n). Ferroptosis inducers such as erastin and RSL3 are ideal probes to elucidate conserved regulators of ferroptosis. RSL3 is a small molecule that can induce ferroptosis by directly binding to GPX4 and blocking the enzyme activity of GPX4 [24, 34], whereas erastin has two individual targets to induce ferroptosis. The first one is targeting system x_c_^-^, which limits cystine/GSH import and inhibits the synthesis of GPX4, causing accumulation of lipid peroxides and ferroptosis. Another is targeting the voltage-dependent anion channels VDAC2/3 and altering the permeability of the outer mitochondrial membrane, which decreases the rate of NADH oxidation [35]. Therefore, erastin-induced ferroptosis is only partially dependent on GPX4 function, but RSL3-induced ferroptosis totally depends on GPX4. Our results showed that LF3 treatment or knockout of TCF4 didn’t promote GC cells sensitivity to RSL3-induced ferroptosis (Supplementary Fig. S5). This further suggests that TCF4 specifically regulates ferroptosis in a GPX4-dependent way.

### The beta-catenin/TCF4 transcription complex promotes GPX4 expression

**Fig. 4:**
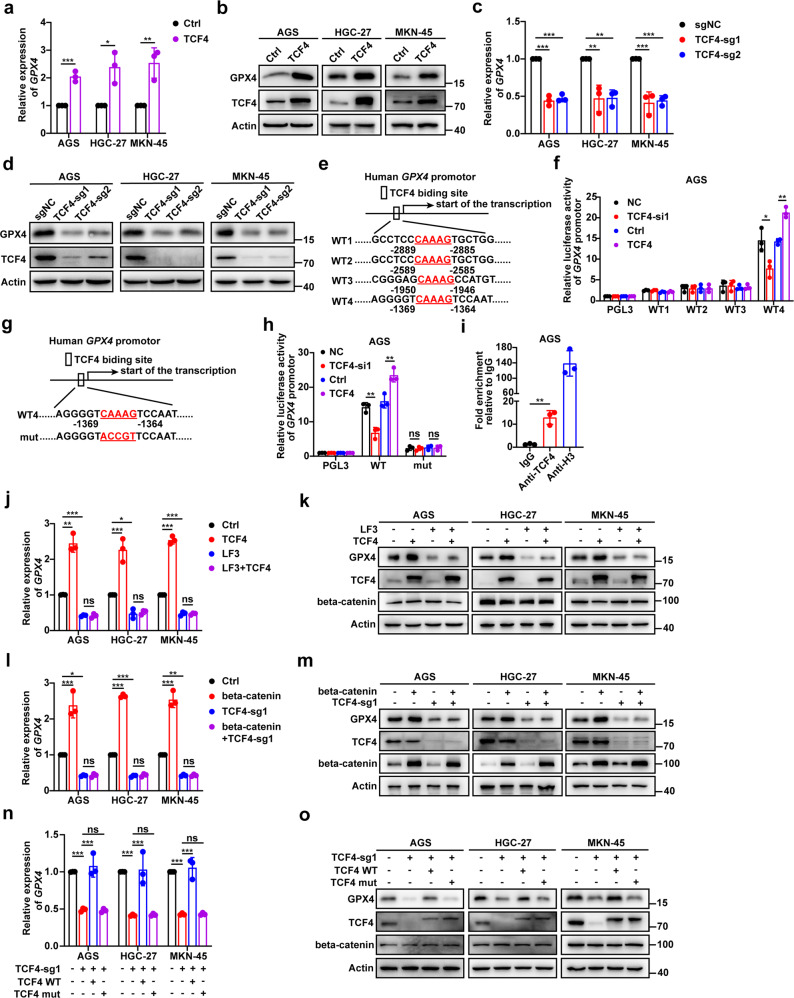
The beta-catenin/TCF4 transcription complex promotes GPX4 expression. **a,** **b** Q-PCR (**a**) and western blot (**b**) analysis of GPX4 mRNA and protein expression in GC cells transfected with TCF4-coding plasmid. **c,** **d** mRNA (**c**) and protein (**d**) expression of GPX4 in GC cells expressing sgNC or sg-TCF4. **e** The four possible TCF4 binding sites in human GPX4 promoter. **f** Transcriptional activity of GPX4 in AGS measured by the luciferase reporter system. **g** The TCF4 binding site in human GPX4 promoter and the corresponding base mutation (WT4, the binding site was intact; mut, the binding site was mutated). **h** Transcriptional activity of GPX4 in TCF4 knockdown or overexpressed AGS measured by the luciferase reporter system. **i** ChIP assay for TCF4 occupancy on the GPX4 promoter. ChIP was performed with chromatin derived from AGS. The final DNA samples were amplified by qPCR with pairs of primers as described in Materials and Methods. A histone H3 antibody was used as a positive control. IgG antibody was used as a negative control. **j**, **k** Q-PCR (**j**) and western blot (**k**) analysis of GPX4 expression in indicated GC cells following treatment with LF3 (AGS and MKN-45 for 10 μΜ, HGC-27 for 2 μΜ) and TCF4-coding plasmid. **l**, **m** mRNA (**l**) and protein (**m**) expression of GPX4 in TCF4-KO GC cells with or without transfection of the beta-catenin-coding plasmid. **n**, **o** Q-PCR (**n**) and western blot (**o**) analysis of GPX4 expression in TCF4-KO GC cells transfected with wild-type or mutant TCF4-coding plasmid (WT: the binding sites of beta-catenin and TCF4 were intact, mut: the binding sites of beta-catenin and TCF4 were mutated). Data are presented as the mean ± SD of three independent experiments. The *p*-values in panels **a**, **f**, **h**, **i** were calculated by Student’s *t*-test. The p-values in panel **c** were calculated by one-way ANOVA. The *p*-values in panels **j**, **l**, **n** were calculated by two-way ANOVA. ns not significant, *p < 0.05, **p < 0.01, ***p < 0.001.

Next, we further explored the specific pattern of TCF4 in the regulation of GPX4. As expected, GPX4 expression was significantly increased upon TCF4 overexpression in GC cells (Fig. 4a, b), while TCF4 knockout reduced its expression (Fig. 4c, d). These results indicated that GPX4 was a putative downstream effector of TCF4-mediated biological functions. According to previous studies, TCF4 promotes the transcription of target genes through the CAAAG sequence [36, 37]. In addition, four classical TCF4 binding motifs were identified in the promoter of GPX4 (3000 bp in front of the transcription start site), and therefore, we constructed four luciferase reporter plasmids containing these motifs (Fig. 4e). TCF4 knockdown could only reduce WT4 luciferase activity, while TCF4 overexpression exhibited the opposite effect, compared with that of WT1, WT2, and WT3 luciferase activities (Fig. 4f and Supplementary Fig. S6a). Then, we constructed luciferase reporter plasmid mutants, in which the TCF4 binding site was mutated (Fig. 4g). The regulation of WT4 luciferase activity by TCF4 knockdown or overexpression was blocked in the mutant plasmid (Fig. 4h and Supplementary Fig. S6b). ChIP assays showed that TCF4 could directly accumulate at GPX4 promoter regions (Fig. 4i and Supplementary Fig. S6c). Taken together, our data indicate that GPX4 is a TCF4 target gene.

Furthermore, we investigated whether TCF4 induces GPX4 expression in association with the Wnt/beta-catenin signaling. Our results showed that GPX4 expression was reduced by beta-catenin knockdown (Supplementary Fig. S6d, e) and the disruption of beta-catenin and TCF4 by LF3 (Fig. 4j, k and Supplementary Fig. S6f). We also found that the induction of GPX4 expression by TCF4 was abrogated by LF3 (Fig. 4j, k and Supplementary Fig. S6f). TCF4 depletion abrogated the induction of GPX4 expression by beta-catenin (Fig. 4l, m and Supplementary Fig. S6g). Then, we constructed a TCF4 mutant plasmid (TCF4 mut) that lacked the beta-catenin binding. The results showed that the induction of GPX4 expression was induced by the TCF4 wild-type plasmid (TCF4 WT) but not with the TCF4 mutant plasmid (Fig. 4n, o and Supplementary Fig. S6h). Therefore, beta-catenin/TCF4 transcription complex promotes GPX4 expression to confer resistance to ferroptosis.

### TCF4 inhibition sensitizes GC cells to cisplatin via ferroptosis induction

**Fig. 5:**
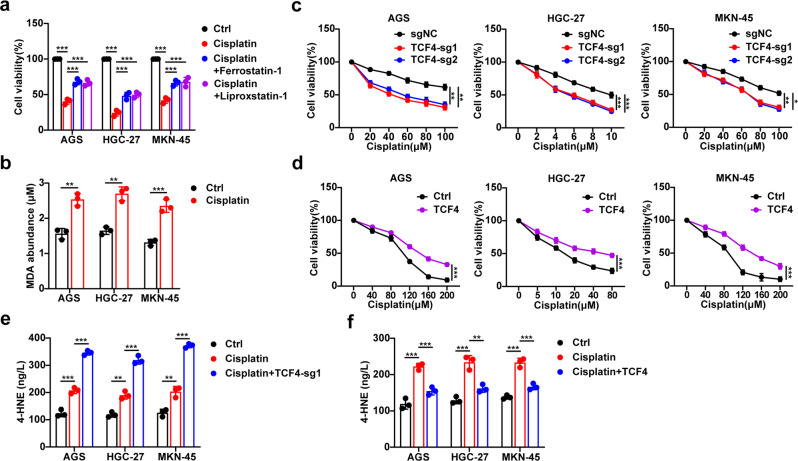
TCF4 inhibition sensitizes GC cells to cisplatin-induced ferroptosis in vitro. **a** Cell viability of indicated GC cells following treatment with cisplatin (AGS and MKN-45 for 100 μM, HGC-27 for 10 μM) and in the absence or presence of ferrostatin-1 (2 μM) or liproxstatin-1 (1 μM) for 24 h. **b** MDA production in GC cells followed by treatment with cisplatin (AGS and MKN-45 for 10 μM, HGC-27 for 1 μM). **c** Cell viability of GC cells expressing sgNC or sg-TCF4 and treated with different concentrations of cisplatin for 24 h. **d** Cell viability of GC cells transfected with control or TCF4-coding plasmid and treated with different concentrations of cisplatin for 24 h. **e**, **f** 4-HNE production in TCF4-KO (**e**) or overexpressing (**f**) GC cells treated with cisplatin (AGS and MKN-45 for 10 μM, HGC-27 for 1 μM). Data are presented as the mean ± SD of three independent experiments. The *p*-values in panels **a**, **e**, **f**, were calculated by one-way ANOVA. The *p*-values in panels **c**, **d**, were calculated by two-way ANOVA. The *p*-values in panel **b** were calculated by Student’s *t*-test. *P < 0.05, **P < 0.01, ***P < 0.001.

Cisplatin-based chemotherapies are the primary treatments for advanced GC, but resistance to cisplatin has become increasingly challenging [7, 8]. Since previous studies showed that ferroptosis can be induced by chemotherapy [28, 29], our results also showed that cisplatin induction of cell death can be partially inhibited by the ferroptosis inhibitor ferrostatin-1 and liproxstatin-1 (Fig. 5a). As expected, we observed an increase in MDA production by cisplatin (Fig. 5b), indicating that cisplatin can induce ferroptosis in GC cells, which is consistent with previous studies [28, 38]. Next, we investigated if TCF4 regulated GC cells sensitivity to cisplatin by ferroptosis. The depletion of TCF4 increased cisplatin-induced cell death (Fig. 5c). Conversely, TCF4 overexpressing GC cells were robustly resistant to cisplatin-induced cell death (Fig. 5d). To validate that cisplatin-induced cell death is ferroptosis, we detected cellular lipid peroxidation level. The production of 4-HNE that is induced by cisplatin was further enhanced in the absence of TCF4, while it was rescued by TCF4 overexpression (Fig. 5e, f). In summary, ferroptosis can be induced by cisplatin in GC cells and TCF4 inhibition sensitizes GC cells to cisplatin-induced ferroptosis in vitro.

**Fig. 6:**
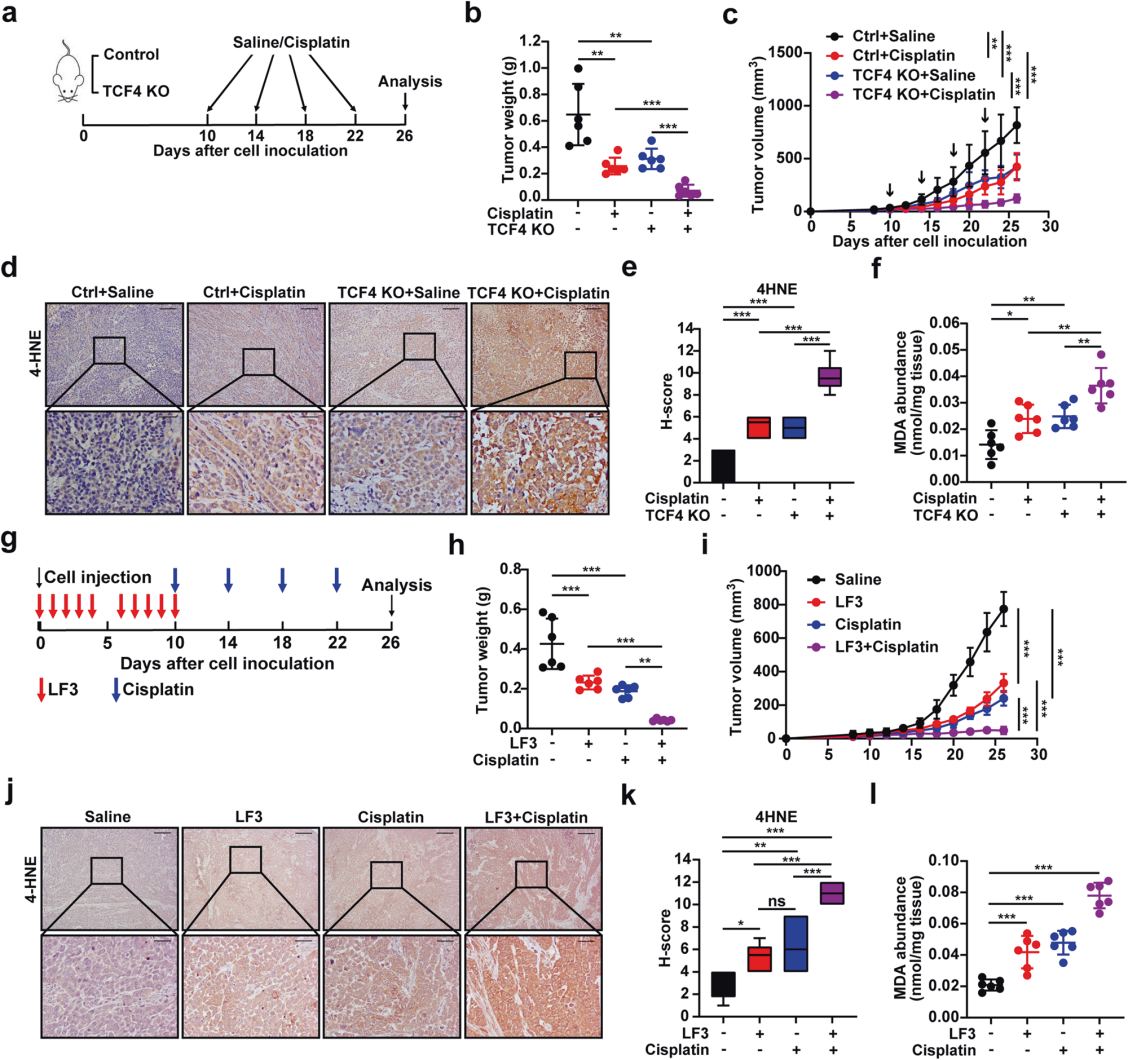
TCF4 deficiency or Wnt signaling inhibition promote cisplatin sensitivity through ferroptosis in vivo. **a** Schematic description of the experimental design used to establish the animal model. **b**, **c** Tumor weight (**b**) and tumor growth curves (**c**) in the nude mouse xenograft model. **d**, **e** IHC staining (**d**) and H-score (**e**) for 4-HNE in xenografts. Scale bars: 200 μm (insets 50 μm). **f** MDA production in tumor tissues. **g** Schematic description of the experimental design used to establish the animal model. **h**, **i**. Tumor weight (**h**) and tumor growth curves (**i**) in the nude mouse xenograft model. **j**, **k** IHC staining (**j**) and H-score (**k**) for 4-HNE in xenografts. Scale bars: 200 μm (insets 50 μm). **l** MDA production in tumor tissues. Data are presented as the mean ± SD of 6 biologically independent animals. All *p*-values were calculated using two-way ANOVA. *p < 0.05, **p < 0.01, ***p < 0.001.

Next, we further investigated in vivo whether controlling the activity of TCF4 can be used as a potential antitumoral therapeutic strategy. For this, we subcutaneously injected TCF4-knockout MKN-45 cells into 6-week male BALB/c nude mice. These tumor-bearing mice were injected with either cisplatin (5 μg/g) or saline every 4 days since day 10. The tumors were harvested on the 26th day and analyzed (Fig. 6a). We found that TCF4 knockout suppressed tumor growth and enhanced sensitivity to cisplatin (Fig. 6b, c and Supplementary Fig. S7a); however, it did not influence mice weight (Supplementary Fig. S7b). The induced production of 4-HNE and MDA by cisplatin was further enhanced in the absence of TCF4 (Fig. 6d–f). Furthermore, the expression of GPX4 was reduced by TCF4 knockout, while the treatment of cisplatin had into effect (Supplementary Fig. S7c, d, e). Taken together, we provide in vivo evidence that demonstrates the role of TCF4 in promoting GPX4 expression, which results in the decrease of lipid peroxidation levels in tumors and facilitates tumor growth.

Then we tested in vivo a pharmacological inhibitor such as LF3 as a potential antitumor therapeutic approach which was more accessible than TCF4-knockout cells. Six-week male BALB/c nude mice were subcutaneously injected with MKN-45 cells and half of them were treated with LF3 at 50 mg/kg [30] or saline by intraperitoneal inoculation ten times since tumor cell injection. The tumor-bearing mice, with or without LF3, were injected with either cisplatin (5 μg/g) or saline every 4 days starting at day 10. The tumors were harvested on the 26th day and analyzed (Fig. 6g). LF3-cisplatin combination showed stronger inhibition than any of the other monotherapies, when starting the treatment after tumors were palpable (Fig. 6h, I and Supplementary Fig. S7f). No systemic toxicity, e.g., weight loss, was observed in the treated mice (Supplementary Fig. S7g). Remarkably, LF3-treated tumors had more lipid peroxidation, as shown by higher levels of 4-HNE and MDA and lower expression of GPX4 (Fig. 6j–l and Supplementary Fig. S7h, i). These data show that LF3-cisplatin combination has potent tumor inhibition capacities in xenotransplanted tumors.

### *H. pylori* promotes cellular lipid peroxidation by upregulating GPX4 expression

**Fig. 7:**
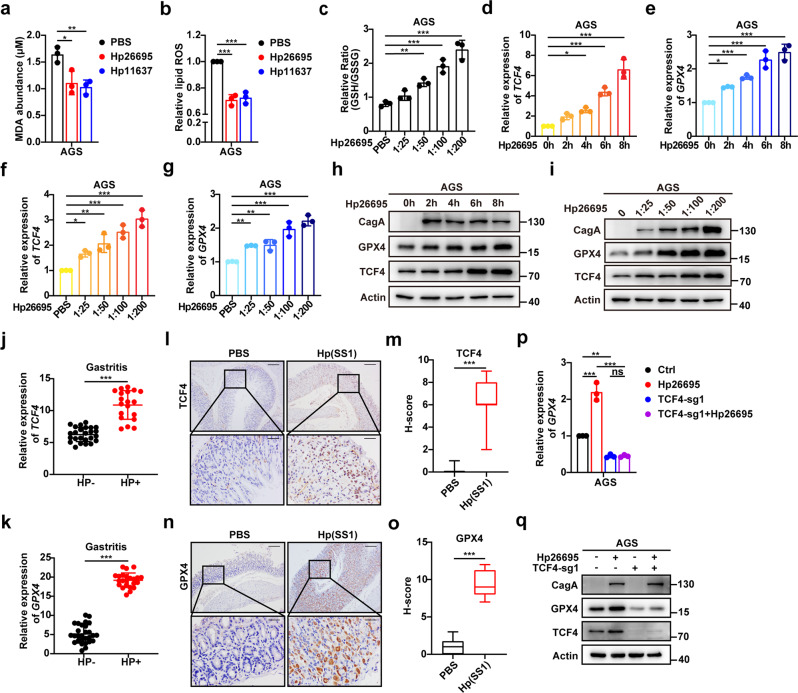
*H. pylori* promotes cellular lipid peroxidation by upregulating GPX4 expression. **a–c** MDA production (**a**), Lipid ROS (**b**), and relative ratio of GSH/GSSG (**c**) in AGS infected with *H. pylori* 26695 or *H. pylori* 11637 for 8 h (MOI = 200). **d**–**g** Q-PCR analysis of TCF4 (**d**, **f**) and GPX4 (**e**, **g**) mRNA expression in AGS infected with *H. pylori* 26695 at different MOI and time points. **h,** **i** Western blot analysis of TCF4 and GPX4 protein expression in AGS infected with *H. pylori* 26695 at different time points (**h**) and MOI (**i**). **j** Q-PCR analysis of TCF4 mRNA expression in *H. pylori*-negative *(n* = 25) or *H. pylori*-positive (*n* = 19) human AG samples. **k** Q-PCR analysis of GPX4 mRNA expression in *H. pylori*-negative (*n* = 28) or *H. pylori*-positive (*n* = 23) human AG samples. **l**, **m** IHC staining (**l**) and H-score (**m**) of TCF4 in normal (*n* = 8) or in *Hp (SSI)*-infected mice (*n* = 8). Scale bars: 200 μm (insets 50 μm). **n**, **o** IHC staining (**n**) and H-score (**o**) of GPX4 in normal (*n* = 8) or in *Hp (SSI)*-infected mice (*n* = 8). Scale bars: 200 μm (insets 50 μm). **p**, **q** Q-PCR (**p**) and western blot (**q**) analysis of GPX4 expression in *H. pylori*-infected TCF4-KO GC cells. Data are presented as the mean ± SD of three independent experiments. The *p*-values in panels **a**–**g** were calculated by one-way ANOVA. The *p*-values in panels **j**, **k**, **m**, **o** were calculated by two tailed unpaired Student’s *t*-test. The *p*-values in panel **p** were calculated by two-way ANOVA. ns not significant, *p < 0.05, **p < 0.01, ***p < 0.001.

Infection by *H. pylori* is a high-risk factor for the occurrence of gastritis and gastric adenocarcinoma [2, 3]. In this study, we observed a significant reduction in lipid ROS and MDA, an increase in the relative ratio of GSH/GSSG in GC cells infected by *H. pylori* 26695 and 11637 (Fig. 7a–c and Supplementary Fig. S8a-c). Both of them were CagA-positive strains. Notably, TCF4 and GPX4 mRNA expressions were induced by *H. pylori* in GC cells infected at different MOI and time points (Fig. 7d–g, and Supplementary Fig. S8d-k). Consistent with the transcriptomic findings, the TCF4 and GPX4 protein expressions were also induced by *H. pylori* infection (Fig. 7h, i and Supplementary Fig. S8l, m). Similarly, TCF4 and GPX4 mRNA levels were higher in *H. pylori*-positive human gastritis samples (Fig. 7j, k). IHC staining indicated that TCF4 and GPX4 levels were higher in the *H. pylori*-infected group when compared with the non-infected control group (Fig. 7l–o). In addition, our results showed that *H. pylori*-mediated upregulation of GPX4 was relieved by TCF4 deficiency, suggesting that TCF4 plays an important role in GPX4 overexpression that is triggered by *H. pylori* (Fig. 7p, q and Supplementary Fig. S8n, o). These results demonstrate that *H. pylori* upregulates GPX4 expression and activity via TCF4, leading to the regulation of lipid peroxidation levels.

## Discussion

Most GC patients are diagnosed at an advanced stage, resulting in a poor overall prognosis. Chemotherapy is one of the main treatments for advanced cancers, and cisplatin is the first-line chemotherapeutic drug [39, 40]; however, resistance to its effect is becoming a therapeutic challenge [38, 41–43]. High concentrations of antioxidants in cancer cells are a considerable obstacle in cancer radiotherapy and chemotherapy [44]. Ferroptosis is an iron-dependent form of regulated cell death caused by the accumulation of lipid peroxidation products, which is prevented by the key antioxidant enzyme GPX4 [21, 24]. Recent studies indicated that the aberrant activation of GPX4 confers resistance to chemotherapy- or radiotherapy-induced ferroptosis [38, 45]. However, the molecular mechanism underlying ferroptosis resistance that is mediated by the aberrant activation of GPX4 in advanced cancers, remains unclear. In the present study, we found that the Wnt/beta-catenin signaling pathway attenuates the levels of cellular lipid peroxidation by upregulating the expression of GPX4, resulting in the inhibition of GC cells’ ferroptosis.

The Wnt/beta-catenin signaling pathway is a conserved signaling axis that participates in diverse physiological processes and is often found abnormally activated in gastrointestinal tumors [46, 47]. Although the Wnt/beta-catenin signaling pathway has been reported to be associated with chemotherapy resistance in various malignancies [48–50], including GC [51], the exact molecular mechanism remains largely unknown. Our data showed that the beta-catenin/TCF4 transcription complex is directly bound to the GPX4 promoter region and promotes its expression which confers ferroptosis resistance. The region from −1467 to −1189 bp upstream of the GPX4 translational starting site has been found to be regulated by the selenium-derived transcriptional activators, TFAP2c and Sp1 [52]. Interestingly, this specific region is also bound by the beta-catenin/TCF4 transcription complex. Thus, our data further imply that the dysregulation of the Wnt/beta-catenin signaling may act as a potential barrier to cancer treatments.

Infection by *H. pylori* is a high-risk factor for the occurrence of gastritis and gastric adenocarcinoma [2, 3]. It is so common that more than 50% of the global population harbors the bacterium. *H. pylori* modulates Wnt/β-catenin-mediated tumorigenesis in human gastric cancer, but the relationship between *H. pylori* and chemotherapy controversial [53, 54]. In our study, we observed that *H. pylori* leads to the high antioxidant status of cancer cells by upregulating GPX4 expression and activity via TCF4. Therefore, eradication of *H. pylori* can relatively enhance chemo-sensitivity in *H. pylori*-positive GC patients by triggering ferroptosis.

It is known that the Wnt/β-catenin signaling is one of the most attractive targets for cancer therapy and a number of Wnt inhibitors have been reported. Although a number of these inhibitors have entered clinical trials, none of these drugs has achieved clinical approval [55]. A major challenge for clinical targeting is to inhibit Wnt signaling in cancer cells while avoiding on-target toxicity in healthy tissues owing to alterations in adult stem cell homeostasis [47, 56, 57]. LF3, a 4-thioureido-benzenesulfonamide derivative, robustly inhibits the interaction between beta-catenin and TCF4, without causing cell death. Remarkably, the population of cells with low Wnt expression did not respond to LF3 treatment [30]. In the present study, we found that LF3 directly targets TCF4 and induces ferroptosis by reducing the expression of GPX4, which is a potential therapeutic strategy to enhance chemo-sensitivity for advanced GC patients.

In summary, our study illustrates a new strategy of ferroptosis regulation through modulating the Wnt/beta-catenin signaling, thereby providing a potential therapeutic strategy to enhance chemo-sensitivity for advanced GC. Finally, ferroptosis has a positive role in radiotherapy and immunotherapy, raising the possibility that targeting Wnt/beta-catenin signaling may also improve the outcomes of those therapies.

## Methods

### Cell culture

AGS and HGC-27 cells were obtained from the Cell bank of Chinese Academy of Science (Shanghai, PR China) and MKN-45 cells from the National infrastructure of Cell Line Resource (Beijing, PR China). AGS cells were cultured in Ham’s F12 medium (HyClone, Logan, UT, USA) with 12% FBS (Gibco, Carlsbad, CA, USA). HGC-27 and MKN-45 cells were cultured in RPMI-1640 (Gibco) with 10% FBS. All cultures were maintained in a humidified 5% CO_2_ incubator (Thermo Fisher Scientific, Waltham, MA, USA) at 37 °C. All cells were authenticated by short tandem repeat profiling and tested free from mycoplasma. A more detailed description of cell lines used in this study was in Supplementary Table S1.

### Reagents and antibodies

Erastin (S7242), ferrostatin-1 (S7243), liproxstatin-1 (S7699), Z-VAD-FMK (S7023), necrosulfonamide (S8251), 3-Methyladenine (S2767), LF3 (S8474), and cisplatin (S1166) were purchased from Selleck Chemicals (Houston, Texas, USA). The stimuli concentrations were as follows: ferrostatin-1, 2 μM; liproxstatin-1, 1 μM; Z-VAD-FMK, 10 μM; necrosulfonamide, 0.5 μM; 3-Methyladenine, 250 μM. Actinomycin D (HY-17559) was purchased from MedChemExpress (Monmouth Junction, NJ, USA). TNF alpha (TNFα, HZ-1014), anti-beta-actin (66009-1-Ig, 1:5000), and anti-GAPDH (60004-1-Ig, 1:5000) were purchased from Proteintech (Wuhan, PR China). Anti-GPX4 (ab125066, 1:2000), anti-beta-catenin (ab32572, 1:1000), and anti-4-HNE (ab46545, 1:100) were purchased from Abcam (Cambridge, UK). Anti-TCF4 (sc-166699, 1:500) for western blotting and anti-CagA (sc-28368, 1:500) were purchased from Santa Cruz Biotechnology (Dallas, Texas, USA). Anti-TCF4 (MA5-32240, 1:100) for IHC was purchased from Thermo Fisher Scientific.

### Colony formation and cell viability assay

For colony formation assays, the cells (500 cells/well) were seeded in a six-well plate and incubated for 1–2 weeks until the appearance of cell colonies, which were fixed with methanol and stained with Giemsa.

Cell viability was assayed by a CCK-8 reagent (HY-K0301, MedChemExpress). In brief, the cells were seeded into 96-well plates and incubated with the indicated treatments. Subsequently, 100 μl of fresh medium was added to cells containing 10 μl of CCK-8 solutions and incubated for 2 h (37 °C, 5% CO_2_). Afterward, the absorbance was measured spectrophotometrically at 450 nm. The collected values were normalized to blank wells and the relative cell viability was normalized to the respective DMSO-treated wells.

### Ethynyldeoxyuridine (EdU) staining

The fraction of DNA-replicating cells, which represents cell proliferation status, was assessed using Cell-Light EdU Apollo488 In Vitro Imaging Kit EdU according to the manufacturer’s instructions (C10310-3, RiboBio, Guangzhou, PR China).

### Wound healing and migration assays

For the wound healing assays, 5 × 10^5^ cells were seeded into a 12-well plate until confluence. Using a pipette tip, a scratch for each culture was performed. After 48 h, accurate wound measurements were taken to calculate the wound closure = (wound width at 0 h − wound width at 48 h)/wound width at 0 h × 100%. The cultures were viewed and photographed using an inverted phase-contrast microscope.

For migration assays, a total of 5 × 10^4^ cells were plated into the upper chamber of a Transwell with polycarbonate filters coated with matrigel (354234, BD Biosciences, Franklin Lakes, NJ, USA). The lower chamber was supplemented with a medium containing 20% FBS. After 48 h, cells that migrated to the lower well were fixed using methanol and stained with crystal violet staining solution.

### Cell death assay

For cell death assay, the cell cultures were treated with different stimuli, collected using trypsin and without EDTA, washed twice with PBS, and resuspended in 100 μl binding buffer with 5 μl propidium iodide (PI) (556463, BD Biosciences) for 5 min. The cells were later analyzed using a CytoFLEX cytometer instrument (Beckman Coulter, Brea, CA, USA) and analyzed by CytExpert software (Beckman Coulter).

### Lipid ROS assay

The cells were plated at 1 × 10^5^ cells per well in a 12-well plate. An amount of 10 μM BODIPY-581/591 C11 (D3861, Thermo Fisher Scientific) was added to the media and incubated for 30 min at 37 °C, 5% CO_2_. The cells were then collected by trypsin without EDTA and washed twice with PBS followed by resuspension in 500 μl PBS. ROS levels were analyzed using a CytoFLEX cytometer instrument (Beckman Coulter), and the data were analyzed by CytExpert software (Beckman Coulter).

### MDA and GSH/GSSG assay

The relative MDA concentration in cell or tumor lysates was assessed using a Lipid Peroxidation (MDA) Assay Kit (ab118970, Abcam), according to the manufacturer’s instructions. This assay measures MDA reaction with thiobarbituric acid (TBA) that generate a MDA-TBA adduct in a sample. The MDA-TBA adduct can be quantified colorimetrically (OD = 532 nm). The ratio of GSH/GSSG was measured according to the manufacturer’s instruction (S0053, Beyotime, Shanghai, PR China).

### 4-HNE assay

ELISA kits (MBS161454, MyBioSource, San Diego, CA, USA) were used to evaluate the concentration of 4-HNE according to the manufacturer’s protocol.

### Cell transfection

Flag-tagged pENTER or pENTER-beta-catenin (Boshang, Jinan, PR China) and Myc-tagged pGV657 or Myc-tagged TCF4 (GENECHEM, Shanghai, PR China) plasmids (2 µg/sample) were transfected into GC cells using the Roche Transfection Reagent (2 µl/sample, Roche, Basel, Switzerland), and as recommended by the manufacturer. The cells were collected 48 h after transfection for further analyses. For transient silencing, siRNA was transfected into GC cells with INTERFERin Reagent (Polyplus-transfection, Illkirch, France) according to the manufacturer’s instructions. For TCF4 transient silencing, the target sequences were as follows: 5’-CGCCAACGACGAACTGATT-3’ (siRNA1) and 5’-CGACAGGAGGATTCAGACA-3’ (siRNA2). For beta-catenin transient silencing, target sequences were as follows: 5’-GCCACAAGATTACAAGAAA-3’ (siRNA1) and 5’-GACTACCAGTTGTGGTTAA-3’ (siRNA2).

### CRISPR-Cas9 assay

The lentivirus carrying TCF4 or GPX4 sgRNA and control sgRNA was purchased from GENECHEM. Knockout cell lines were generated by lentivirus infection and puromycin selection according to the manufacturer’s instructions. The target sequences of TCF4 were as follows: AGCAATGAACACTTCACGCC (sgRNA1) and AATACGGGGATATATCTGGA (sgRNA2). The target sequence of GPX4 was AGCCCCGCCGCGATGAGCCT.

### RNA isolation and real-time quantitative polymerase chain reaction (qRT-PCR)

Total RNA was extracted using Trizol reagent (Invitrogen, Carlsbad, CA, USA). The concentration and quality of RNA were determined using a NanoDrop one spectrophotometer (Thermo Fisher Scientific). An amount of 1 μg of RNA was used to synthesize cDNA using the RT reagent Kit gDNA Eraser (RR047A, Takara, Shiga, Japan). The corresponding cDNAs were subjected to quantitative PCR analysis using TB Green (RR820A, Takara). The used primers for qRT-PCR assays are listed in Supplementary Table S2.

### Immunohistochemistry (IHC) and quantification

FFPE (formalin-fixed, paraffin-embedded) sections acquired from patients or mice tissues were deparaffinated, dehydrated and subjected to antigen retrieval by placing them in citrate buffer in a 98 °C water bath for 15 min to shelter endogenous peroxidase activity. The samples were also subjected to H_2_O_2_ treatment and blocked with a 3% BSA solution for 30 min. Thereafter, the samples were incubated with specific primary antibodies overnight at 4 °C. On the following day, sections were incubated with corresponding secondary antibodies and then analyzed using a DAB staining kit (Vector Laboratories, Burlingame, CA, USA). The intensity of positive staining was scored by “H-score” as follows: 0 (no staining); 1 (slightly brown); 2 (moderately brown), and 3 (dark brown). A scale from 0 to 3 was used to score the proportion of positively stained cells: 0 (0%), 1 (<25%), 2 (25–75%), and 3 (>75%). The H-score was calculated as a multiple of the above two scores.

### Western blot

Total proteins were extracted with RIPA lysis buffer (Beyotime) containing protease inhibitors, and the protein concentrations were detected with BCA Protein Assay Kit (Thermo Fisher Scientific). SDS-PAGE was used to separate the proteins that were transferred afterward onto PVDF membranes (Millipore, Bedford, MA, USA). The membranes were blocked with 5% non-fat milk for 2 h at room temperature and incubated overnight at 4 °C with primary antibodies. This step was followed by incubations with secondary antibodies. The signals were detected with ECL detection reagent (Millipore) according to standard protocols. Full-length original western blots in this article are provided in Supplementary File.

### Dual-luciferase reporter gene assay

Human GPX4 WT1, WT2, WT3, and WT4 promoter fragments were synthesized by GENEray Biotechnology (Shanghai, PR China). The KOD-PlusMutagenesis Kit (TOYOBO, Osaka, Japan) was used to mutate the binding sites and the mutation was confirmed by DNA sequencing. The Luciferase Assay System (Promega, Madison, WI, USA) was used to measure luciferase reporter activity.

### Chromatin immunoprecipitation (ChIP) assay, ChIP-seq, and data analysis

For the ChIP assay, the SimpleChIP^®^ Enzymatic Chromatin IP Kit (56383, Cell Signaling Technology, Danvers, MA, USA) was used according to the manufacturer’s protocol. ChIP-seq and data analysis were completed by Aksomics (Shanghai, PR China). For each sample, 10 ng DNA from each sample was sequenced by Illumina NovaSeq 6000, according to the manufacturer’s instructions. The sequence quality of the raw data was examined using the FastQC software. The clean reads passed a Solexa CHASTITY quality filter that was aligned to the mouse reference genome UCSC MM10 by BOWTIE (V2.1.0). MACS V1.4.2 was used for peak calling of the ChIP regions. Statistically significant ChIP-enriched regions (peaks) were identified by comparison of IP and Input, using a *p*-value threshold of 10^−4^. The peaks were annotated by the nearest genes using the newest UCSC RefSeq database, and these genes were analyzed by Metascape.

### *H. pylori* cultures and *H. pylori*-infected mouse model

All animal experiments were reviewed and approved by The Ethical Committee of School of Basic Medical Sciences, Shandong University (reference number: 2017-2-003). *H. pylori* strains 26695 and 11637 were grown in Brucella broth supplemented with 5% FBS under microaerophilic conditions (5% O_2_, 10% CO_2_, and 85% N_2_) at 37 °C. *H. pylori*-infected mouse model was structured as previously described [58].

### Human clinical specimens

Samples of GC and adjacent non-tumor tissues were collected from Shandong Tumor Hospital, while samples of atrophic gastritis (AG) with *H. pylori*-positive or negative were obtained from Jinan Central Hospital, Shandong, PR China. The diagnosis of *H. pylori* infection in AG patients was performed by ^13^C urea breath test. IHC samples including adjacent non-tumor tissues, SG (superficial gastritis), AG with IM (atrophic gastritis with intestinal metaplasia), dysplasia, and GC, were acquired from Bengbu Medical University (Anhui, PR China). The study protocol was approved by Shandong University Research Ethics Committee.

### Animal studies

All mice were maintained under SPF housing and with a maximum of five mice per cage. In the first vivo experiment, 6-week-old male BALB/c nude mice were obtained from Vital River Laboratory Animal Technology (Beijing, PR China). TCF4-KO MKN-45 cells (4 × 10^5^) and control cells were subcutaneously injected into 24 nude mice, respectively. On day 10 after injection and when palpable tumors were present, the control group or TCF4-KO group mice were divided into two groups randomly by weight. These tumor-bearing mice were intraperitoneally injected with either cisplatin (5 μg/g) or saline every 4 days. Tumor growth was monitored every 2 days and the mice were culled after 26 days. In the second in vivo experiment, 24 6-week male BALB/c nude mice were subcutaneously injected with MKN-45 cells, and half of them were treated with LF3 at 50 mg/kg [30] or saline by intraperitoneal inoculation for ten times since tumor cell injection. Then, tumor-bearing mice with or without LF3 were injected with either cisplatin (5 μg/g) or saline every 4 days starting at day 10. The tumors were harvested on the 26th day and analyzed. Experiments were blinded to the person performing marker analysis.

### Statistical analysis

All experiments were repeated at least three times. Data were presented as mean ± standard deviation. All the performed statistical analyses are described in each figure legend. Statistical *p*-values were obtained by application of the appropriate statistical tests using the GraphPad Prism 8. For all tests, *p* < 0.05 was considered significant (ns: not significant, *p < 0.05, **p < 0.01, ***p < 0.001).

## Supplementary information

Pre Authorship Form

Reproducibility Checklist

Supplementary Figure S1

Supplementary Figure S2

Supplementary Figure S3

Supplementary Figure S4

Supplementary Figure S5

Supplementary Figure S6

Supplementary Figure S7

Supplementary Figure S8

Supplementary Figure Legends

Supplementary Table S1

Supplementary Table S2

Original File of Western Blots

## Data Availability

The survival curves of GPX4 were derived from GEPIA (http://gepia.cancer-pku.cn/). The survival curves of TCF4 were derived from The Kaplan–Meier plotter (http://kmplot.com/analysis/). The GPX4 and TCF4 gene expression data in human cancer tissues were derived from TCGA database. All the data supporting the findings of this study are available from the corresponding author on reasonable request.
