# Supplementary material for: Wnt/beta-catenin signaling confers ferroptosis resistance by targeting GPX4 in gastric cancer
Source: Cell Death Differ. 2022 May 9;29(11):2190–202. doi: 10.1038/s41418-022-01008-w (PMC9613693; doi:10.1038/s41418-022-01008-w)
Supplement: Supplementary file 1 — Pre Authorship Form [file 41418_2022_1008_MOESM1_ESM.pdf]

SPRINGER NATURE

Change of authorship request form - Journals (pre-acceptance)

Important information. Please read.

- This form should be used by authors to request any change in authorship (adding/deleting authors) including changes in corresponding authors. This form should not be used for name changes. Please fully complete all sections. Use black ink and block capitals and provide each author's full name with the given name first followed by the family name.

- By signing this declaration, all authors guarantee that the order of the authors are in accordance with their scientific contribution, if applicable as different conventions apply per discipline, and that only authors have been added who made a meaningful contribution to the work.

- Please note, in author collaborations where there is formal agreement for representing the collaboration, it is sufficient for the representative or legal guarantor (usually the corresponding author) to complete and sign the Authorship Change Form on behalf of all authors, **next to the added/removed author(s)**. **(Complete Section 3, followed by Section 6.)**

  In author collaborations where there is no formal agreement for representing the collaboration and **there are more than 10 authors**, one may sign for all, provided the signer appends correspondence that attests that each of the authors have agreed to the change **and the added/removed authors sign the form**. **(Complete Section 3, followed by Section 6.)**

- Please note, we cannot investigate or mediate any authorship disputes. If you are unable to obtain agreement from all authors (including those who you wish to be removed) you must refer the matter to your institution(s) for investigation. Please inform us if you need to do this.

- If you are not able to return a fully completed form within **30 days** of the date that it was sent to the author requesting the change, we may have to withdraw your manuscript. We cannot publish manuscripts where authorship has not been agreed by all authors (including those who have been removed).

- Incomplete forms will be rejected.

- Please return/upload this form, fully completed, to the Journals Editorial Office. The Journal and/or Publisher will consider the information you have provided to decide whether to approve the proposed change in authorship. We may decide to contact your institution for more information or undertake a further investigation, if appropriate, before making a final decision.

SPRINGER NATURE Springer Nature is one of the world's leading global research, educational and professional publishers, created in May 2015 through the combination of Nature Publishing Group, Palgrave Macmillan, Macmillan Education and Springer Science+Business Media. ---

SPRINGER NATURE

Change of authorship request form - Journals (pre-acceptance)

Section 1: Please provide the current title of manuscript

Manuscript ID no.: CDD-21-2337RRRR

Title: Wnt/beta-catenin signaling confers ferroptosis resistance by targeting GPX4 in gastric cancer

Section 2: Please provide the previous authorship, in the order shown on the manuscript before the changes were introduced. Please indicate the corresponding author by adding (CA) behind the name.

	First name(s)	Family name	ORCID or SCOPUS id, if available
1st author	YUE	WANG	
2nd author	LIXIN	ZHENG	
3rd author	WENJING	SHANG	
4th author	ZONGCHENG	YANG	
5th author	TONGYU	LI	
6th author	FEN	LIU	
7th author	WEI	SHAO	
8th author	LIN	LV	
9th author	LI	CHAI	
10th author	LINGXIN	QU	

Please use an additional sheet if there are more than 10 authors.

Additional sheet of Section 2

11th author	QING	XU	
12th author	JIE	DU	
13th author	JIPING (CA)	ZENG (CA)	0000-0002-3212-7853
14th author	JIHUI (CA)	JIA (CA)	0000-0002-5529-8735

SPRINGER NATURE Springer Nature is one of the world's leading global research, educational and professional publishers, created in May 2015 through the combination of Nature Publishing Group, Palgrave Macmillan, Macmillan Education and Springer Science+Business Media. ---

Section 3: Please provide a justification for change. Please use this section to explain your reasons for changing the authorship of your manuscript, e.g. what necessitated the change in authorship? Please refer to the (journal) policy pages for more information about authorship. Please explain why omitted authors were not originally included and/or why authors were removed on the submitted manuscript.

We add a contributing author Xiuming Liang in the manuscript for his constructive directions duiring the period of manuscript revision. Dr.Liang cooperated with our lab, designed part of the experiments and gave financial support to this research. Dr.Liang was mainly involved in the manuscript revision process, so we omitted him in the original author list.

Section 4: Proposed new authorship. Please provide your new authorship list in the order you would like it to appear on the manuscript. Please indicate the corresponding author by adding (CA) behind the name. If the Corresponding Author has changed, please indicate the reason under section 3.

	First name(s)	Family name (this name will appear in full on the final publication and will be searchable in various abstract and indexing databases)	Affiliated institute	E-mail address
1st author	YUE	WANG	1 Department of Microbiology/Key Laboratory for Experimental Teratology of the Chinese Ministry of Education, School of Basic Medical Science, Cheeloo College of Medicine, Shandong University, Jinan, Shandong, PR China; 2 Key Laboratory of Infection and Immunity of Shandong Province, School of Basic Medical Science, Cheeloo College of Medicine, Shandong University, Jinan, Shandong, PR China; 3 Shandong University-Karolinska Institute Collaborative Laboratory for Cancer Research, School of Basic Medical Science, Cheeloo College of Medicine, Shandong University, Jinan, Shandong, PR China	15650585212@126.com
2nd author	LIXIN	ZHENG	1 Department of Microbiology/Key Laboratory for Experimental Teratology of the Chinese Ministry of Education, School of Basic Medical Science, Cheeloo College of Medicine, Shandong University, Jinan, Shandong, PR China; 2 Key Laboratory of Infection and Immunity of Shandong Province, School of Basic Medical Science, Cheeloo College of Medicine, Shandong University, Jinan, Shandong, PR China	zhenglixin555@163.com
3rd author	WENJING	SHANG	Shandong University-Karolinska Institute Collaborative Laboratory for Cancer Research, School of Basic Medical Science, Cheeloo College of Medicine, Shandong University, Jinan, Shandong, PR China	17865136693@163.com

Please use an additional sheet if there are more than 10 authors.

Additional sheet of Section 4 is in the next page.

Additional sheet of Section 4:

	First name(s)	Family name (this name will appear in full on the final publication and will be searchable in various abstract and indexing databases)	Affiliated institute	E-mail address
4th author	ZONGCHENG	YANG	Shandong University-Karolinska Institute Collaborative Laboratory for Cancer Research, School of Basic Medical Science, Cheeloo College of Medicine, Shandong University, Jinan, Shandong, PR China	yang_chen1107@163.com
5th author	TONGYU	LI	Shandong University-Karolinska Institute Collaborative Laboratory for Cancer Research, School of Basic Medical Science, Cheeloo College of Medicine, Shandong University, Jinan, Shandong, PR China	lty63910@163.com
6th author	FEN	LIU	1 Department of Microbiology/Key Laboratory for Experimental Teratology of the Chinese Ministry of Education, School of Basic Medical Science, Cheeloo College of Medicine, Shandong University, Jinan, Shandong, PR China; 2 Key Laboratory of Infection and Immunity of Shandong Province, School of Basic Medical Science, Cheeloo College of Medicine, Shandong University, Jinan, Shandong, PR China	lf6093@126.com
7th author	WEI	SHAO	1 Department of Microbiology/Key Laboratory for Experimental Teratology of the Chinese Ministry of Education, School of Basic Medical Science, Cheeloo College of Medicine, Shandong University, Jinan, Shandong, PR China; 2 Key Laboratory of Infection and Immunity of Shandong Province, School of Basic Medical Science, Cheeloo College of Medicine, Shandong University, Jinan, Shandong, PR China	shaoweicharlotte@outlook.com
8th author	LIN	LV	1 Department of Microbiology/Key Laboratory for Experimental Teratology of the Chinese Ministry of Education, School of Basic Medical Science, Cheeloo College of Medicine, Shandong University, Jinan, Shandong, PR China; 2 Key Laboratory of Infection and Immunity of Shandong Province, School of Basic Medical Science, Cheeloo College of Medicine, Shandong University, Jinan, Shandong, PR China	525181726@qq.com
9th author	LI	CHAI	1 Department of Microbiology/Key Laboratory for Experimental Teratology of the Chinese Ministry of Education, School of Basic Medical Science, Cheeloo College of Medicine, Shandong University, Jinan, Shandong, PR China; 2 Key Laboratory of Infection and Immunity of Shandong Province, School of Basic Medical Science, Cheeloo College of Medicine, Shandong University, Jinan, Shandong, PR China	17865190715@163.com
10th author	LINGXIN	QU	1 Department of Microbiology/Key Laboratory for Experimental Teratology of the Chinese Ministry of Education, School of Basic Medical Science, Cheeloo College of Medicine, Shandong University, Jinan, Shandong, PR China; 2 Key Laboratory of Infection and Immunity of Shandong Province, School of Basic Medical Science, Cheeloo College of Medicine, Shandong University, Jinan, Shandong, PR China	17865191682@163.com
11th author	QING	XU	1 Department of Microbiology/Key Laboratory for Experimental Teratology of the Chinese Ministry of Education, School of Basic Medical Science, Cheeloo College of Medicine, Shandong University, Jinan, Shandong, PR China; 2 Key Laboratory of Infection and Immunity of Shandong Province, School of Basic Medical Science, Cheeloo College of Medicine, Shandong University, Jinan, Shandong, PR China	972018568@qq.com
12th author	JIE	DU	1 Department of Microbiology/Key Laboratory for Experimental Teratology of the Chinese Ministry of Education, School of Basic Medical Science, Cheeloo College of Medicine, Shandong University, Jinan, Shandong, PR China; 2 Key Laboratory of Infection and Immunity of Shandong Province, School of Basic Medical Science, Cheeloo College of Medicine, Shandong University, Jinan, Shandong, PR China	17853141970@163.com

Additional sheet of Section 4:

	First name(s)	Family name (this name will appear in full on the final publication and will be searchable in various abstract and indexing databases)	Affiliated institute	E-mail address
13th author	XIUMING	LIANG	1 Shandong University-Karolinska Institute Collaborative Laboratory for Cancer Research, School of Basic Medical Science, Cheeloo College of Medicine, Shandong University, Jinan, Shandong, PR China;<br>2 Biomolecular Medicine, Clinical Research Center, Department of Laboratory Medicine, Karolinska Institute, Stockholm, Sweden	liangxm@sdu.edu.cn
14th author	JIPING (CA)	ZENG (CA)	Department of Biochemistry and Molecular Biology, School of Basic Medical Sciences, Cheeloo College of Medicine, Shandong University, Jinan, Shandong, PR China	zengjp@sdu.edu.cn
15th author	JIHUI (CA)	JIA (CA)	1 Department of Microbiology/Key Laboratory for Experimental Teratology of the Chinese Ministry of Education, School of Basic Medical Science, Cheeloo College of Medicine, Shandong University, Jinan, Shandong, PR China; 2 Key Laboratory of Infection and Immunity of Shandong Province, School of Basic Medical Science, Cheeloo College of Medicine, Shandong University, Jinan, Shandong, PR China; 3 Shandong University-Karolinska Institute Collaborative Laboratory for Cancer Research, School of Basic Medical Science, Cheeloo College of Medicine, Shandong University, Jinan, Shandong, PR China	jiajihui@sdu.edu.cn

SPRINGER NATURE

Change of authorship request form - Journals (pre-acceptance)

Section 5: Author contribution, Acknowledgement and Disclosures. Please use this section to provide a new disclosure statement and, if appropriate, acknowledge any contributors who have been removed as authors and ensure you state what contribution any new authors made (if applicable per the journal or book (series) policy). Please ensure these are updated in your manuscript - after approval of the change(s) - as our production department will not transfer the information in this form to your manuscript.

New acknowledgements:

Not applicable.

New Disclosures (financial and non-financial interests, funding):

This work was supported by the National Natural Science Foundation of China (Nos. 82172284, 81772151, 81971901, 82002107, 81871620, 81801983 and 81871627).

New Author Contributions statement (if applicable per the journal policy):

Y.W., L.Z., X.L., J.Z. and J.J. conceived the study, designed the experiments, and provided direction. W.Y., L.Z., W.S., Z.Y., T.L., F.L., W.S., L.L., L.C., L.Q., Q.X. and J.D. did the experiments. Y.W., Z.Y. and W.S. analyzed the data. Y.W. wrote the paper. J.Z. and J.J. supervised the study. L.Z., X.L., J.Z. and J.J. provided financial support.

State 'Not applicable' if there are no new authors.

SPRINGER NATURE Springer Nature is one of the world's leading global research, educational and professional publishers, created in May 2015 through the combination of Nature Publishing Group, Palgrave Macmillan, Macmillan Education and Springer Science+Business Media. ---

SPRINGER NATURE

Change of authorship request form - Journals (pre-acceptance)

Section 6: Declaration of agreement. *All* authors, unchanged, new and removed *must* sign this declaration.
(NB: Please print the form, (docu)-sign and return/upload a scanned copy. Please note that signatures that have been inserted as an image file are acceptable as long as it is handwritten. Typed names in the signature box are unacceptable.) *** Please delete as appropriate. Delete all of the bold if you were on the original authorship list and are remaining as an author.**

	First name	Family name		Signature	Date
1st author	YUE	WANG	I agree to the proposed new authorship shown in section 4	YUE WANG	2022-04-09
2nd author	LIXIN	ZHENG	I agree to the proposed new authorship shown in section 4	LIXIN ZHENG	2022-04-09
3rd author	WENJING	SHANG	I agree to the proposed new authorship shown in section 4 d	WEN JING SHANG	2022-04-09
4th authors	ZONGCHENG	YANG	I agree to the proposed new authorship shown in section 4	ZONG CHENG YANG	2022-04-09
5th author	TONGYU	LI	I agree to the proposed new authorship shown in section 4	TONG YU LI	2022-04-09
6th author	FEN	LIU	I agree to the proposed new authorship shown in section 4	FEN LIU	2022-04-09
7th author	WEI	SHAO	I agree to the proposed new authorship shown in section 4	WEI SHAO	2022-04-09

SPRINGER NATURE Springer Nature is one of the world's leading global research, educational and professional publishers, created in May 2015 through the combination of Nature Publishing Group, Palgrave Macmillan, Macmillan Education and Springer Science+Business Media.

SPRINGER NATURE

Change of authorship request form - Journals (pre-acceptance)

	First name	Family name		Signature	Date
8th author	LIN	LV	I agree to the proposed new authorship shown in section 4	LIN LV	2022-04-09
9th author	LI	CHAI	I agree to the proposed new authorship shown in section 4	LI CHAI	2022-04-09
10th author	LINGXIN	QU	I agree to the proposed new authorship shown in section 4	LINGXIN QU	2022-04-09

Please use an additional sheet if there are more than 10 authors.

Additional sheet of Section 6 is in the next page.

In case of author collaborations with formal agreement:

	Name of consortium/consortia	First name	Family name		Signature	Date
Representative/legal guarantor				I agree to the proposed new authorship shown in section 4 /**and the addition/removal*of my name to the authorship list** /and the proposed change in corresponding author		

Both added/removed authors should complete the information in the first table under Section 6.

---- End of form ----

SPRINGER NATURE Springer Nature is one of the world's leading global research, educational and professional publishers, created in May 2015 through the combination of Nature Publishing Group, Palgrave Macmillan, Macmillan Education and Springer Science+Business Media. ---

Additional sheet of Section 6

	First name	Family name		Signature	Date
11th author	QING	XU	I agree to the proposed new authorship shown in section 4	QING XU	2022-04-09
12th authors	JIE	DU	I agree to the proposed new authorship shown in section 4	JIE DU	2022-04-09
13th author	XIUMING	LIANG	I agree to the proposed new authorship shown in section 4 and the addition of my name to the authorship list	XIUMING LIANG	2022-04-09
14th author	JIPING	ZENG	I agree to the proposed new authorship shown in section 4	JIPING ZENG	2022-04-09
15th author	JIHUI	JIA	I agree to the proposed new authorship shown in section 4	JIHUI JIA	2022-04-09
