# Supplementary figures and images for: Wnt/beta-catenin signaling confers ferroptosis resistance by targeting GPX4 in gastric cancer
Source: Cell Death Differ. 2022 May 9;29(11):2190–202. doi: 10.1038/s41418-022-01008-w (PMC9613693; doi:10.1038/s41418-022-01008-w)

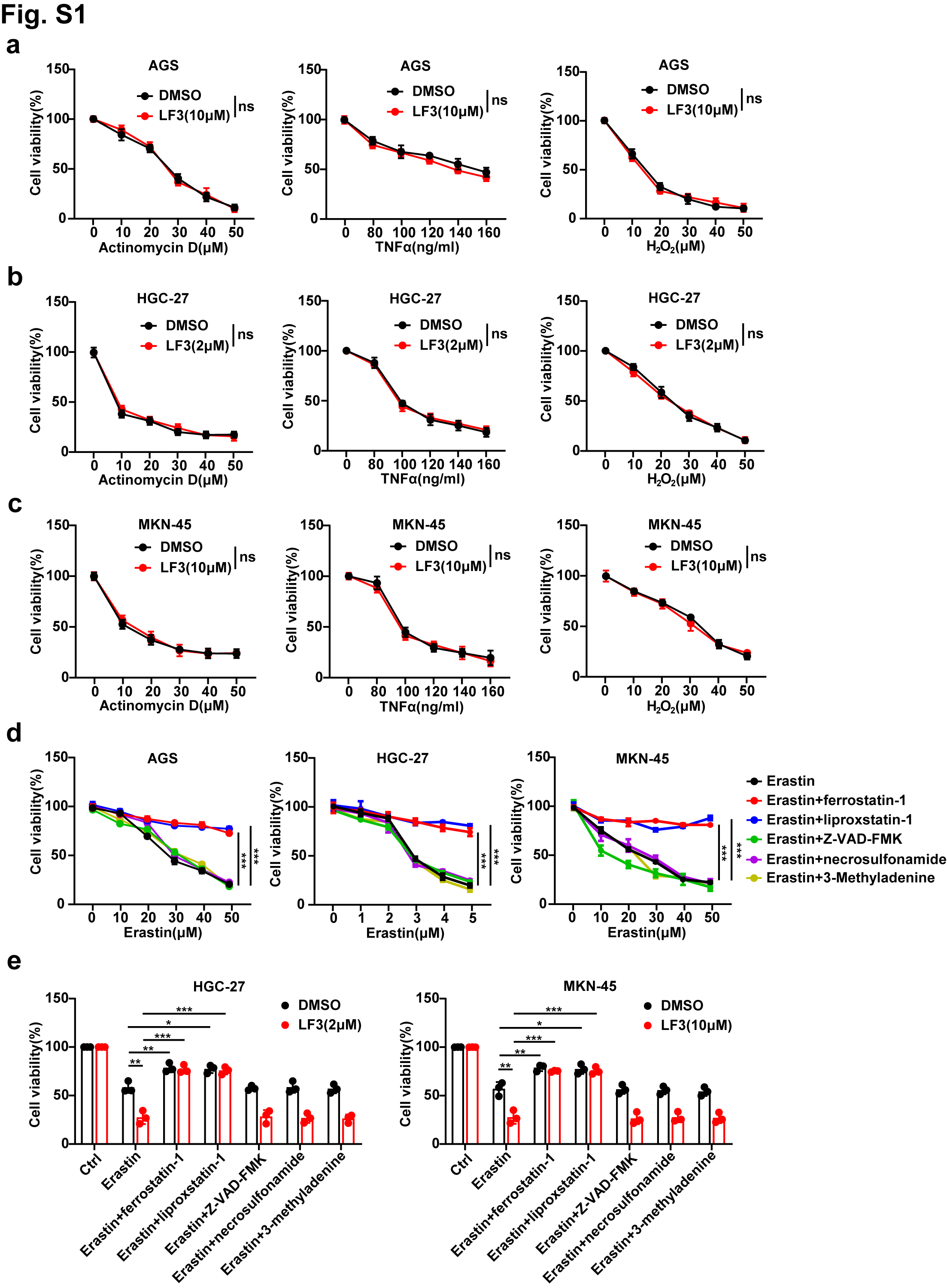

Supplement: Supplementary file 3 — Supplementary Figure S1 [file 41418_2022_1008_MOESM3_ESM.tif]

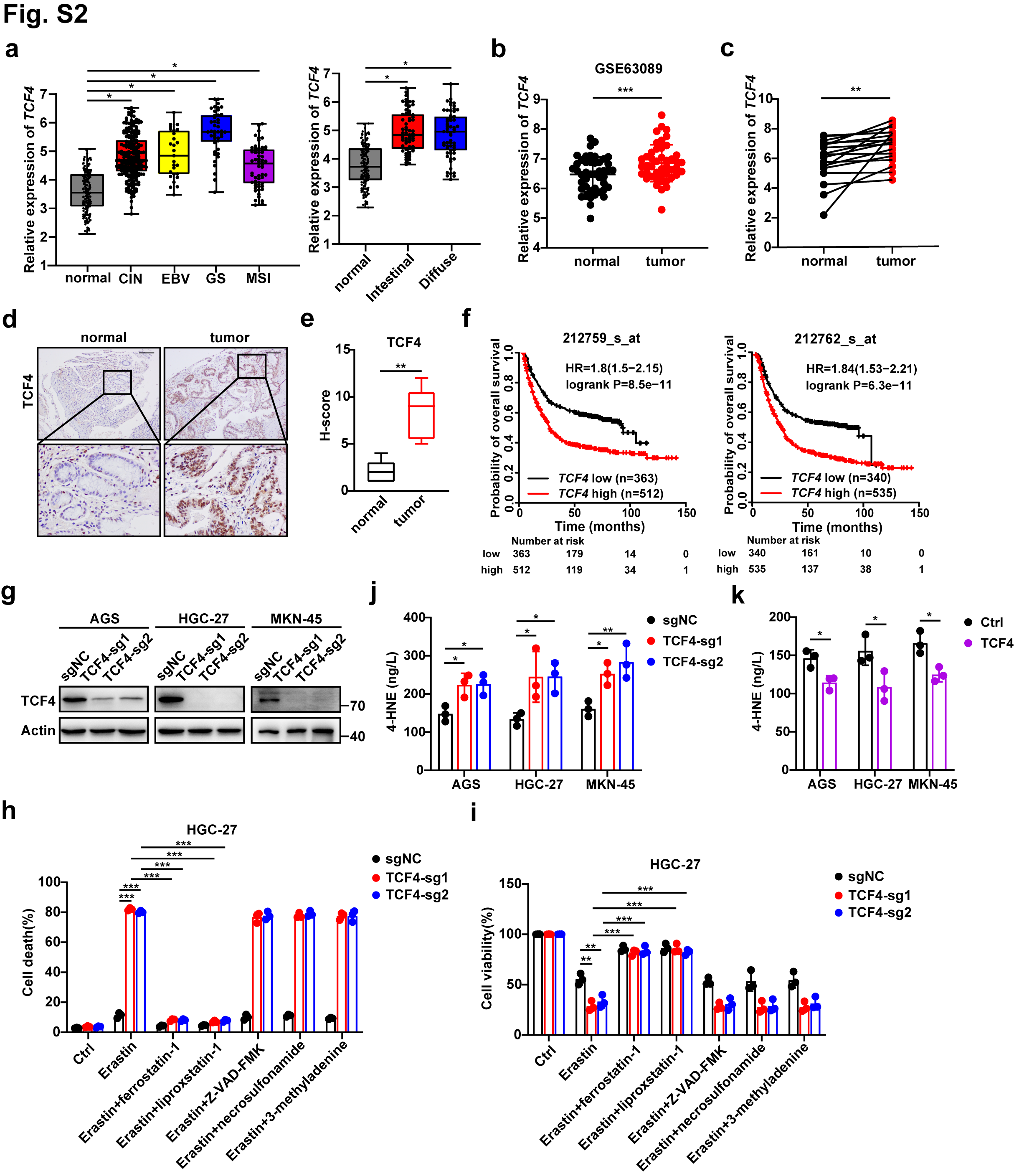

Supplement: Supplementary file 4 — Supplementary Figure S2 [file 41418_2022_1008_MOESM4_ESM.tif]

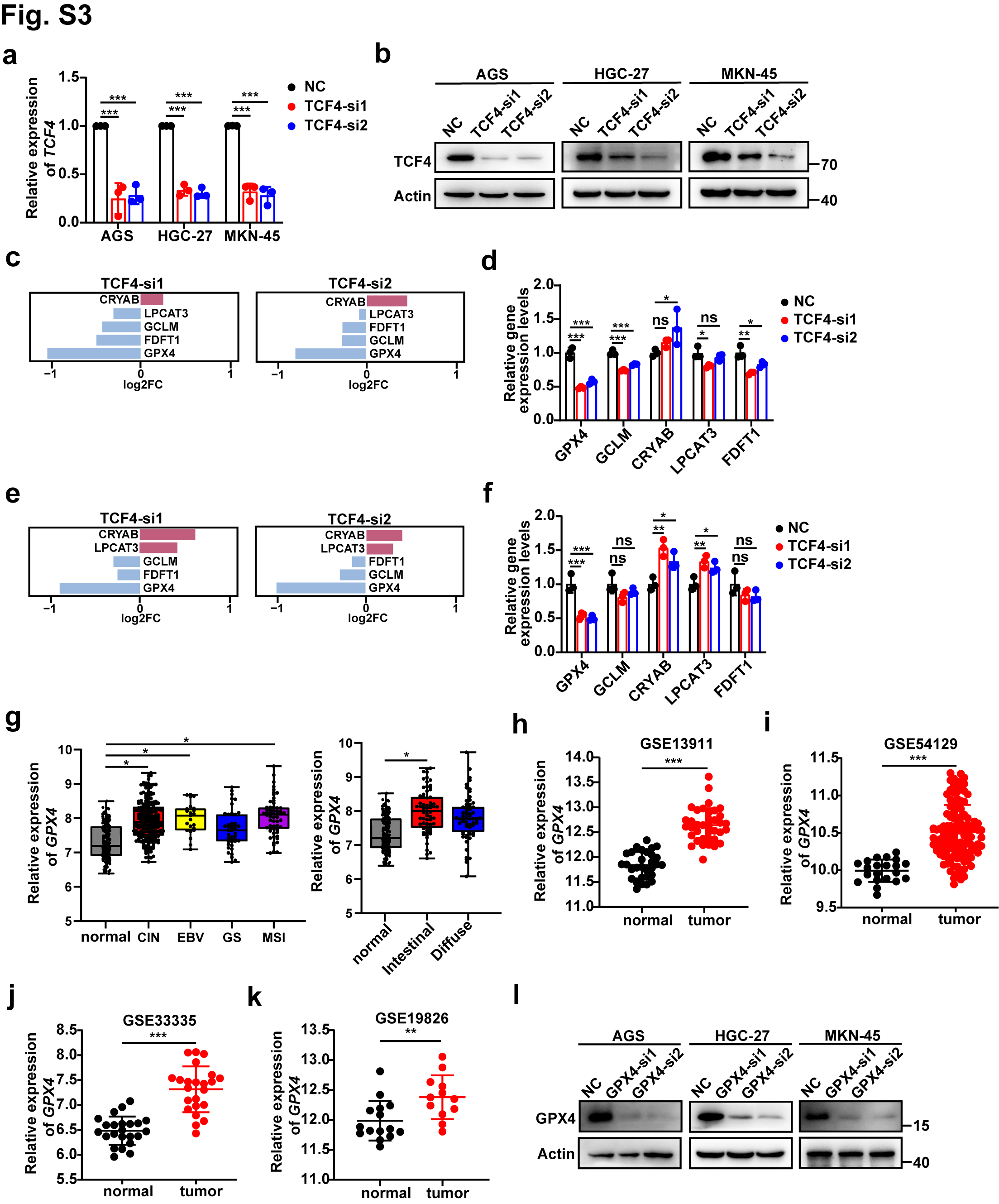

Supplement: Supplementary file 5 — Supplementary Figure S3 [file 41418_2022_1008_MOESM5_ESM.tif]

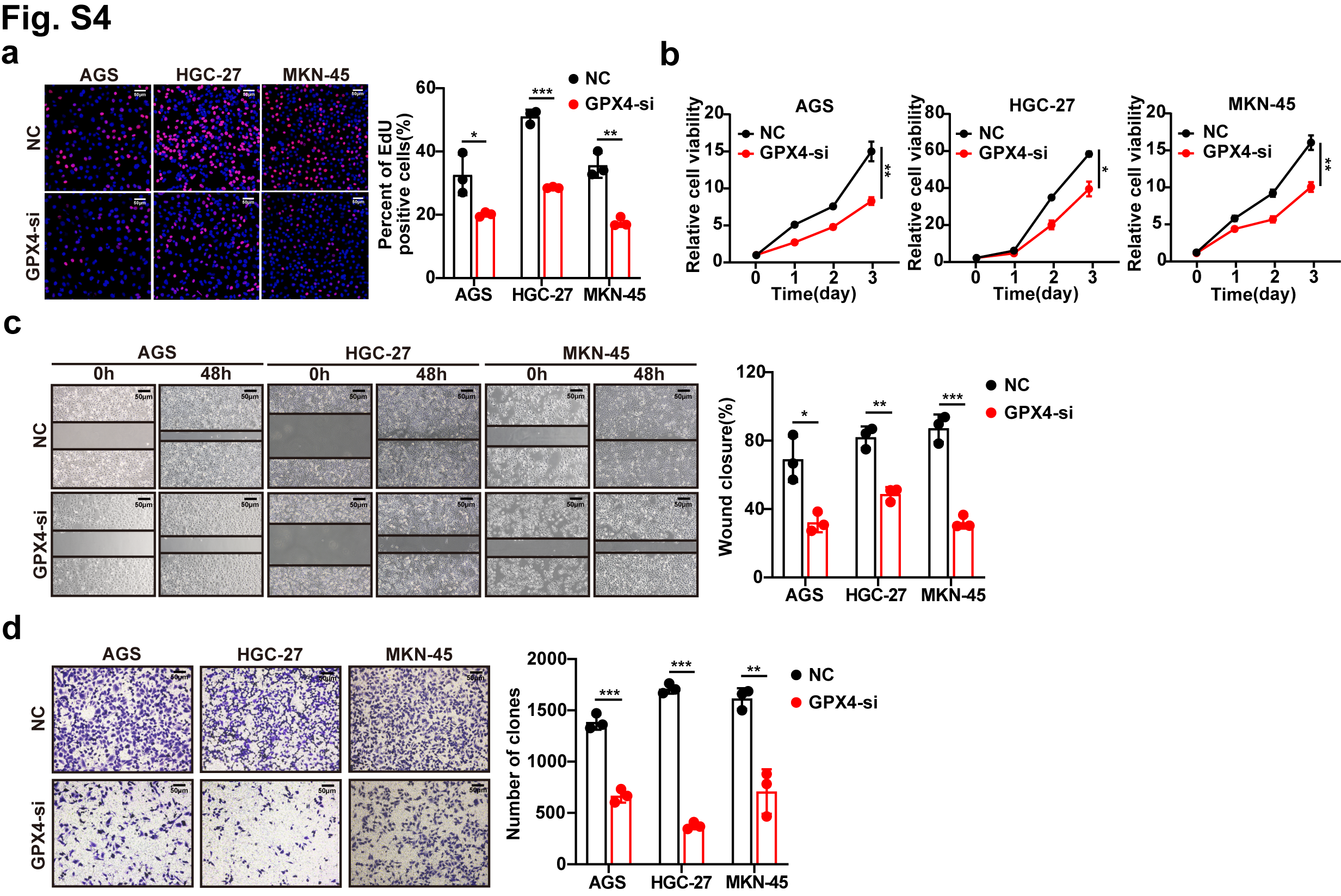

Supplement: Supplementary file 6 — Supplementary Figure S4 [file 41418_2022_1008_MOESM6_ESM.tif]

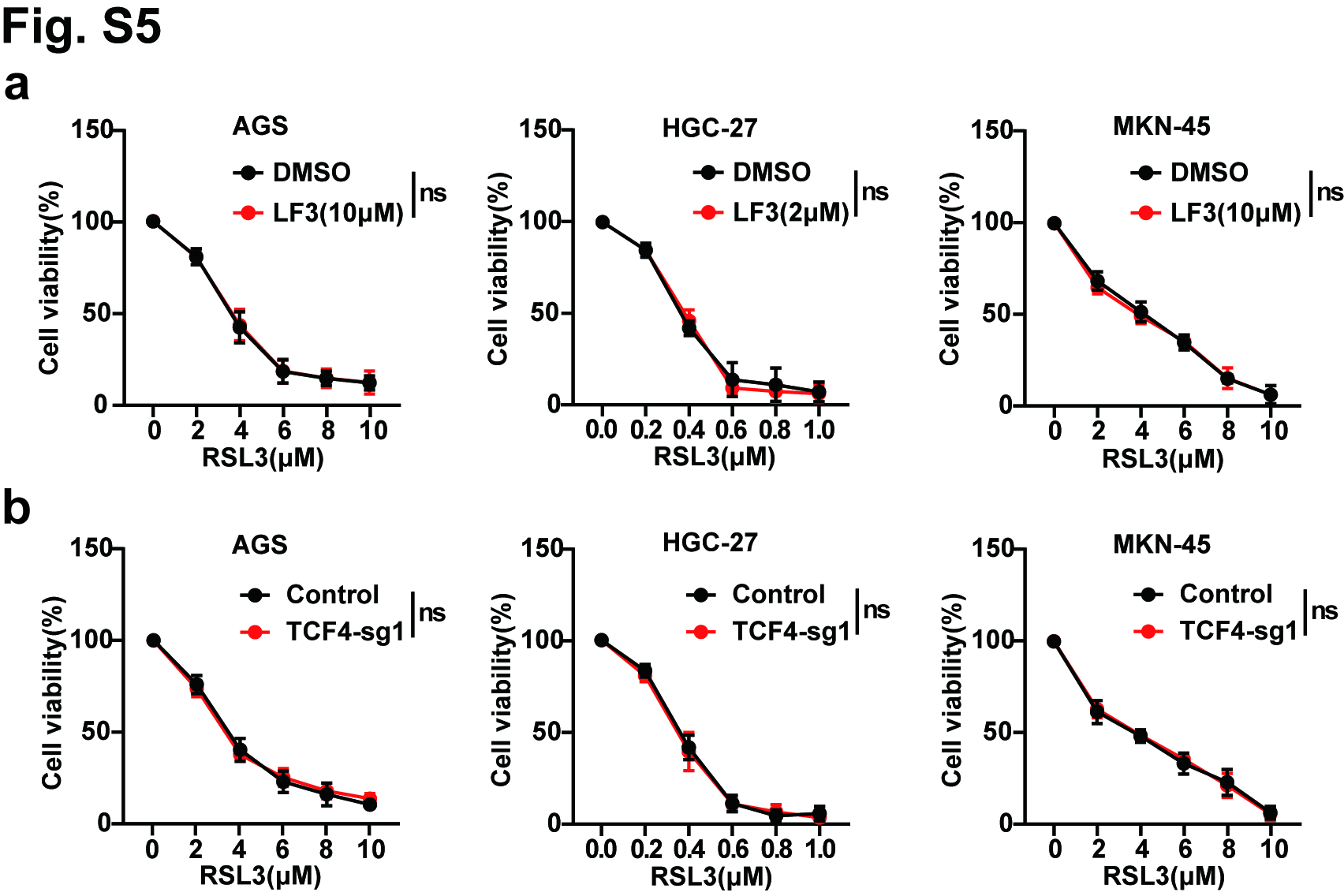

Supplement: Supplementary file 7 — Supplementary Figure S5 [file 41418_2022_1008_MOESM7_ESM.tif]

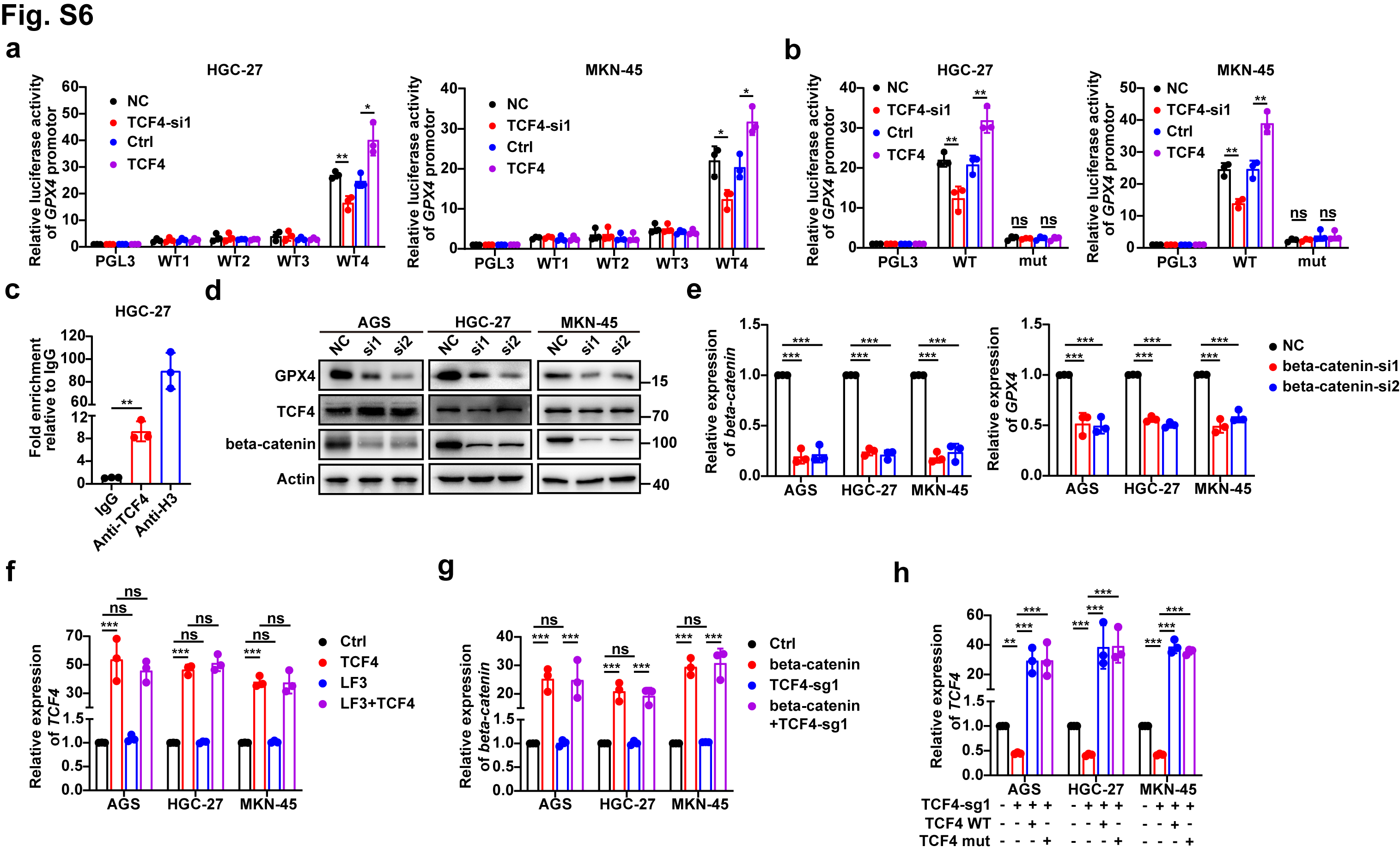

Supplement: Supplementary file 8 — Supplementary Figure S6 [file 41418_2022_1008_MOESM8_ESM.tif]

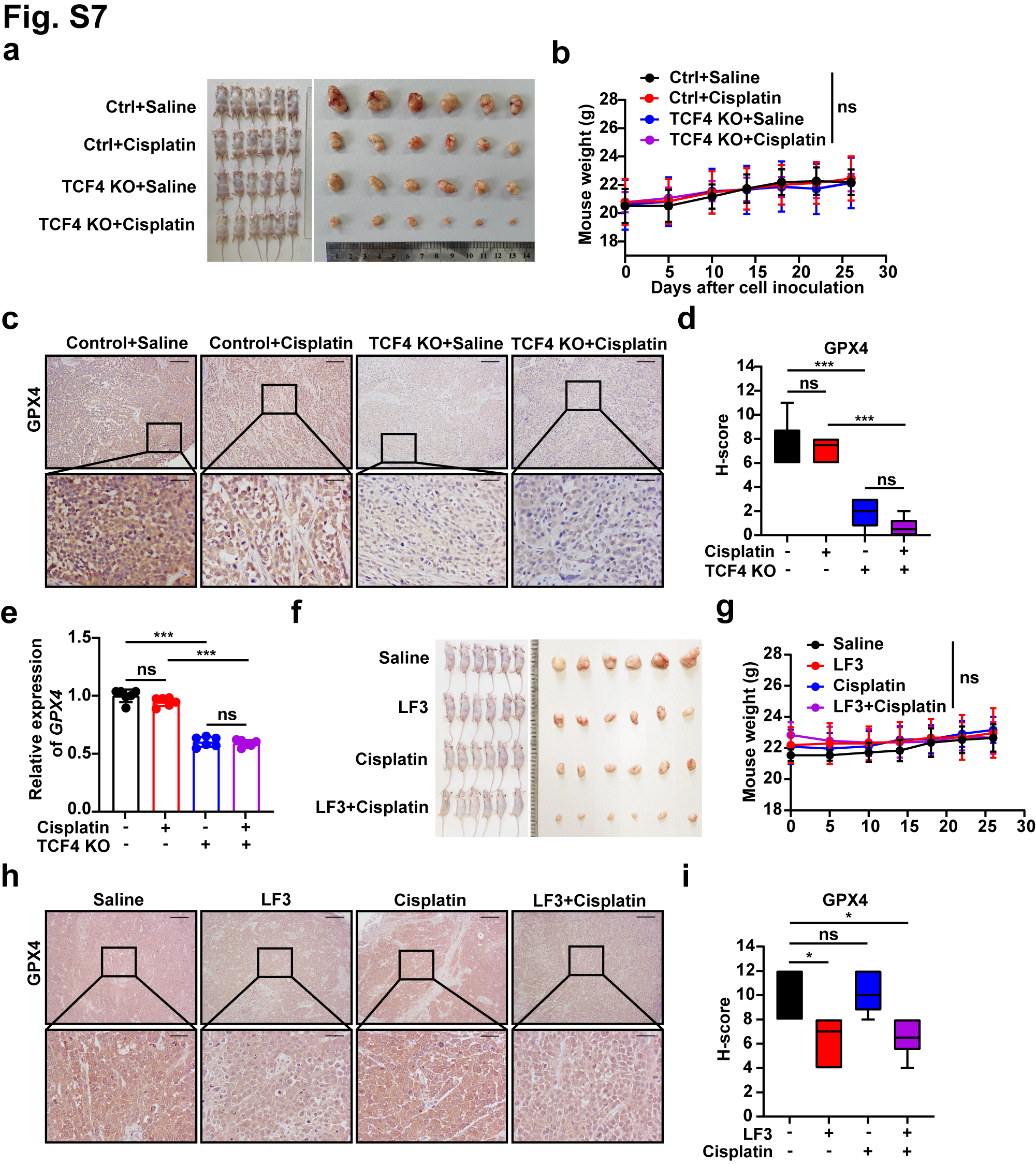

Supplement: Supplementary file 9 — Supplementary Figure S7 [file 41418_2022_1008_MOESM9_ESM.tif]

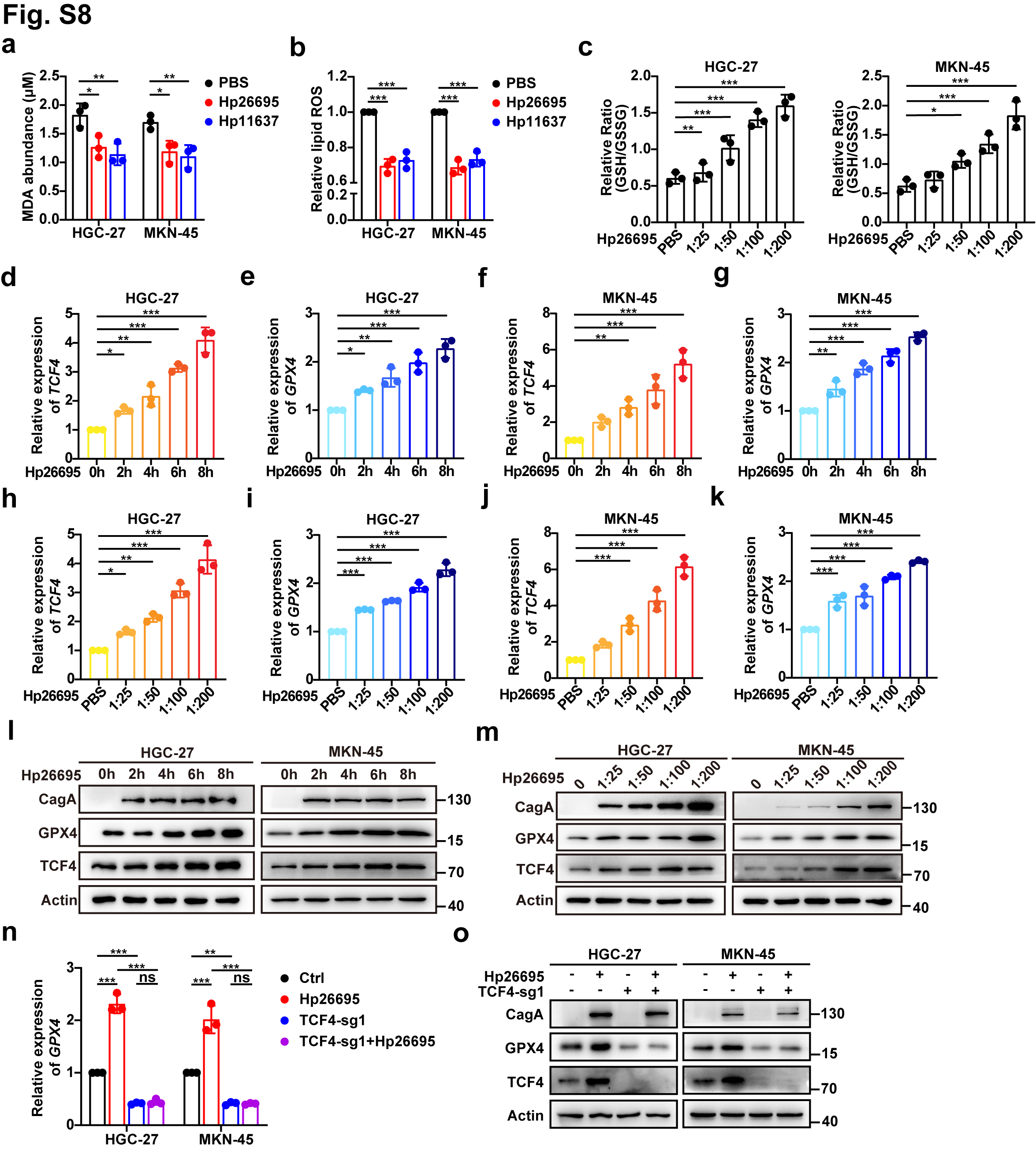

Supplement: Supplementary file 10 — Supplementary Figure S8 [file 41418_2022_1008_MOESM10_ESM.tif]
