# Supplementary material for: Wnt/beta-catenin signaling confers ferroptosis resistance by targeting GPX4 in gastric cancer
Source: Cell Death Differ. 2022 May 9;29(11):2190–202. doi: 10.1038/s41418-022-01008-w (PMC9613693; doi:10.1038/s41418-022-01008-w)
Supplement: Supplementary file 11 — Supplementary Figure Legends [file 41418_2022_1008_MOESM11_ESM.docx]

**Supplementary Figure Legends**

**Supplementary Fig. S1** **Inhibition of the Wnt/beta-catenin signaling enhances GC cells’ sensitivity to ferroptosis.** **a-c** Cell viability of indicated GC cells treated with actinomycin D (**a**), TNFα (**b**) or H_2_O_2_ (**c**) in the absence or presence of LF3 (AGS and MKN-45 for 10 μΜ, HGC-27 for 2 μΜ) for 24 h. **d** Cell viability of indicated GC cells following treatment with erastin and in the absence or presence of ferrostatin-1 (2 μM), liproxstatin-1 (1 μM), Z-VAD-FMK (10 μM), or necrosulfonamide (0.5 μM) or 3-methyladenine (250 μM) for 24 h. **e** Cell viability of indicated GC cells following treatment with erastin (HGC-27 for 3 μM and MKN-45 for 20 μM) in the absence or presence of ferrostatin-1 (2 μM), liproxstatin-1 (1 μM), Z-VAD-FMK (10 μM), necrosulfonamide (0.5 μM) or 3-methyladenine (250 μM) for 24 h. Data are presented as the mean ± SD of three independent experiments. The *p*-values in Supplementary Fig. S1a-d were calculated by two-way ANOVA. The *p*-values in Supplementary Fig. S1e were calculated by one-way ANOVA. ns: not significant, *P<0.05, **P<0.01, ***p < 0.001.

**Supplementary Fig. S2** **TCF4 expression is elevated in GC and associated with poor clinical outcome.** **a** The Cancer Genome Atlas (TCGA) analysis of stomach adenocarcinoma for TCF4 mRNA expression in different subtypes of GC tissues and normal tissues. **b** TCF4 mRNA expression in paired GC tissues and adjacent normal tissues in GEO dataset GSE 63089 (n=45). **c** Q-PCR analysis of TCF4 mRNA expression in paired GC tissues and adjacent normal tissues (n=18). **d** and **e** IHC staining (**d**) and H-score (**e**) for TCF4 in paired GC tissues and adjacent normal tissues (n=5). Scale bars: 200 μm (insets 50 μm). **f** The overall survival for GC patients was analyzed using Kaplan-Meier curves with two different probes (log-rank test; n=875). **g** TCF4 protein expression in sgNC or sg-TCF4 expressing GC cells. **h** Cell death measurement of HGC-27 expressing sgNC or sg-TCF4 treated with erastin (2 μM) in the absence or presence of ferrostatin-1 (2 μM), liproxstatin-1 (1 μM), Z-VAD-FMK (10 μM), necrosulfonamide (0.5 μM) or 3-methyladenine (250 μM) for 24 h. **i** Cell viability of HGC-27 expressing sgNC or sg-TCF4 treated with erastin (3 μM) in the absence or presence of ferrostatin-1 (2 μM), liproxstatin-1 (1 μM), Z-VAD-FMK (10 μM), necrosulfonamide (0.5 μM) or 3-methyladenine (250 μM) for 24 h. **j** 4-HNE production in GC cells expressing sgNC or sg-TCF4. **k** 4-HNE production in GC cells transfected with control or TCF4-coding plasmid. Data are presented as the mean ± SD of three independent experiments. The *p*-values in Supplementary Fig. S2a, j were calculated by one-way ANOVA. P values in Supplementary Fig. S2h, i were calculated by two-way ANOVA. The *p*-values in Supplementary Fig. S2b, c, e, k were calculated by Student’s t-tests. *P<0.05, **P<0.01, ***P<0.001.

**Supplementary Fig. S3** **GPX4 is highly expressed in GC tissues. a** and **b** Q-PCR (**a**) and western blot (**b**) analysis of TCF4 expression in TCF4 siRNA transfected GC cells. **c** and **e** Bar chart showing log2FC of the expression of five genes in TCF4 siRNA transfected AGS and MKN-45 cells. **d** and **f** Relative gene expression levels of GPX4, GCLM, CRYAB, LPCAT3, and FDFT1. **g** The Cancer Genome Atlas (TCGA) analysis of stomach adenocarcinoma for GPX4 mRNA expression in different subtypes of GC tissues and normal tissues. **h-k** TCF4 mRNA expression in GC tissues and adjacent normal tissues in the GEO datasets, GSE 13911 (n=67) (**h**), GSE 54129 (n=131) (**i**), GSE 33335 (n=46) (**j**) and GSE 19826 (n=27) (**k**). **l** GPX4 protein expression of GPX4 in GPX4 siRNA transfected GC cells. Data are presented as the mean ± SD of three independent experiments. The *p*-values in Supplementary Fig. S3a, c-g were calculated by one-way ANOVA. The *p*-values in Supplementary Fig. S3h-k were calculated by Student’s t-tests. ns: not significant, *P<0.05, **P<0.01, ***P<0.001.

**Supplementary Fig. S4** **GPX4 promotes tumorigenesis and metastasis in GC cells.** **a** EdU staining analysis of DNA replication of GPX4 siRNA transfected GC cells. Scale bars: 50 μm. **b** Relative cell viability of GPX4 siRNA transfected GC cells. **c** Transwell penetration assays in GC cells transfected with GPX4 siRNA. Scale bars: 50 μm. **d** Wound healing assays in GPX4 siRNA transfected GC cells. Scale bars: 50 μm. Data are presented as the mean ± SD of three independent experiments. The *p*-values in Supplementary Fig. S4a, c, d, were calculated by Student’s t-tests. The *p*-values in Supplementary Fig. S3b were calculated by two-way ANOVA. *p < 0.05, ***p < 0.001.

**Supplementary Fig. S5 Inhibition or knockout of TCF4 cannot promote RSL3-induced ferroptosis in a GPX4 inactivation manner.** **a** Cell viability of indicated GC cells following treatment with RSL3 in the absence or presence of LF3 (AGS and MKN-45 for 10 μΜ, HGC-27 for 2 μΜ) for 24 h. **b** Cell viability of GC cells expressing sgNC or sg-TCF4 treated with different concentrations of RSL3 for 24 h. Data are presented as the mean ± SD of three independent experiments. The *p*-values were calculated by two-way ANOVA. ns: not significant.

**Supplementary Fig. S6** **The beta-catenin/TCF4 transcription complex promotes GPX4 expression. a** Transcriptional activity of GPX4 in GC cells measured by the luciferase reporter system. **b** Transcriptional activity of GPX4 in TCF4 knockdown or overexpressed GC cells measured by the luciferase reporter system. **c** ChIP assay for TCF4 occupancy on the GPX4 promoter. ChIP was performed with chromatin derived from HGC-27. The final DNA samples were amplified by qPCR with pairs of primers as described in Materials and Methods. A histone H3 antibody was used as a positive control. IgG was used as a negative control. **d** and **e** Protein (**d**) and mRNA (**e**) expression of GPX4 in beta-catenin siRNA transfected GC cells. **f** TCF4 mRNA expression in indicated GC cells following treatment with LF3 (AGS and MKN-45 for 10 μΜ, HGC-27 for 2 μΜ) and TCF4-coding plasmid. **g** Q-PCR analysis of beta-catenin mRNA expression in TCF4-KO GC cells with or without transfection of the beta-catenin-coding plasmid. **h** Q-PCR analysis of TCF4 expression in TCF4-KO GC cells transfected with wild type or mutant TCF4-coding plasmid (WT: the binding sites of beta-catenin and TCF4 were intact, mut: the binding sites of beta-catenin and TCF4 were mutated). Data are presented as the mean ± SD of three independent experiments. The *p*-values in Supplementary Fig. S6a, b, e, were calculated by one-way ANOVA. The *p*-value in Supplementary Fig. S6c was calculated by Student’s t-tests. The *p*-values in Supplementary Fig. S6f-h were calculated by two-way ANOVA. ns: not significant, *p < 0.05, **p < 0.01, ***p < 0.001.

**Supplementary Fig. S7** **TCF4 deficiency or Wnt signaling inhibition promotes cisplatin sensitivity through ferroptosis in vivo.** **a** Images of tumors in each group. **b** Mouse weight in the nude mouse xenograft model. **c** and **d** IHC staining (**c**) and H-score (**d**) for GPX4 in xenografts. Scale bars: 200 μm (insets 50 μm). **e** Q-PCR analysis of GPX4 mRNA expression in tumor tissues. **f** Images of tumors in each group. **g** Mouse weight in the nude mouse xenograft model. **h** and **i** IHC staining (**h**) and H-score (**i**) for GPX4 in xenografts. Scale bars: 200 μm (insets 50 μm). Data are presented as the mean ± SD of 6 biologically independent animals. All *p*-values were calculated by two-way ANOVA. ns: not significant, *p < 0.05, ***p < 0.001.

**Supplementary Fig. S8** ***H. pylori* promotes cellular lipid peroxidation by upregulating GPX4 expression.** **a-c** MDA production (**a**), Lipid ROS (**b**) and relative ratio of GSH/GSSG (**c**) in GC cells infected with *H. pylori* 26695 or *H. pylori* 11637 for 8 h (MOI=200). **d-k** Q-PCR analysis of TCF4 (**d**, **f**, **h**, **j**) and GPX4 (**e**, **g**, **i**, **k**) mRNA expression in GC cells infected with *H. pylori* 26695 at different MOI and time points. **l** and **m** Western blot analysis of TCF4 and GPX4 protein expression in GC cells infected with *H. pylori* 26695 at different time points (**l**) and MOI (**m**). **n** and **o** Q-PCR (**n**) and western blot (**o**) analysis of GPX4 expression in *H. pylori* infected TCF4-KO GC cells. Data are presented as the mean ± SD of three independent experiments. The *p*-values in Supplementary Fig. 87a-k were calculated by one-way ANOVA. The *p*-values in Supplementary Fig. S8n were calculated by two-way ANOVA. ns: not significant, *p < 0.05, **p < 0.01, ***p < 0.001.
