# Supplementary material for: Wnt/beta-catenin signaling confers ferroptosis resistance by targeting GPX4 in gastric cancer
Source: Cell Death Differ. 2022 May 9;29(11):2190–202. doi: 10.1038/s41418-022-01008-w (PMC9613693; doi:10.1038/s41418-022-01008-w)
Supplement: Supplementary file 12 — Supplementary Table S1 [file 41418_2022_1008_MOESM12_ESM.docx]

**Supplementary Table S1: Description of cell lines used in the study**

| Name | AGS | HGC-27 | MKN-45 |
| --- | --- | --- | --- |
| Disease Subtype | Gastric adenocarcinoma | Gastric adenocarcinoma | Gastric adenocarcinoma |
| Primary/Metastasis | Primary | Metastasis from lymph node | Metastasis from liver |
| Sex of cell | Female | Sex unspecified | Female |
| Age at sampling | 54 | Age unspecified | 62 |
| Species of origin | Homo sapiens | Homo sapiens | Homo sapiens |
| Morphology | Epithelioid | Epithelioid | Epithelioid |
| TP53 mutation | No | Yes | Yes |
| CDH1 mutation | Yes | No | Yes |
| Previous therapies | No | No | No |
