# Supplementary material for: Wnt/beta-catenin signaling confers ferroptosis resistance by targeting GPX4 in gastric cancer
Source: Cell Death Differ. 2022 May 9;29(11):2190–202. doi: 10.1038/s41418-022-01008-w (PMC9613693; doi:10.1038/s41418-022-01008-w)
Supplement: Supplementary file 13 — Supplementary Table S2 [file 41418_2022_1008_MOESM13_ESM.docx]

**Supplementary Table S2: qRT-PCR primers used in the study**

| Name | Sequence |
| --- | --- |
| *β-actin* | Forward: 5′-AGTTGCGTTACACCCTTTCTTG-3′ |
|  | Reverse: 5′-CACCTTCACCGTTCCAGTTTT-3′ |
| *β-2M* | Forward: 5′-GAATTGCTATGTGTCTGGGT-3′ |
|  | Reverse: 5′-CATCTTCAAACCTCCATGATG-3′ |
| *TCF4* | Forward: 5′-GCCTCTTATCACGTACAGCAAT-3′ |
|  | Reverse: 5′-GCCAGGCGATAGTGGGGTAAT-3′ |
| *GPX4* | Forward: 5′-TGGGAAATGCCATCAAGTGG-3′ |
|  | Reverse: 5′-GGTCCTTCTCTATCACCAGGGG-3′ |
| *β-catenin* | Forward: 5′-AAAGCGGCTGTTAGTCACTGG-3′ |
|  | Reverse: 5′-CGAGTCATTGCATACTGTCCAT-3′ |
| *CRYAB* | Forward: 5′-CCTGAGTCCCTTCTACCTTCG-3′ |
|  | Reverse: 5′-CACATCTCCCAACACCTTAACTT-3′ |
| *LPCAT3* | Forward: 5′-GGAGCTGAGCCTTAACAAGTT-3′ |
|  | Reverse: 5′-CAAAGCAAAGGGGTAACCCAG-3′ |
| *GCLM* | Forward: 5′-TGTCTTGGAATGCACTGTATCTC-3′ |
|  | Reverse: 5′-CCCAGTAAGGCTGTAAATGCTC-3′ |
| *FDFT1* | Forward: 5′-CCACCCCGAAGAGTTCTACAA-3′ |
|  | Reverse: 5′-TGCGACTGGTCTGATTGAGATA-3′ |
| ChIP | Forward: 5′-GTGGCAACAGAAACACTCATCTC-3′ |
|  | Reverse: 5′-CCAGCTACTCAGGAGACTGAGGCAGGA-3′ |
